# Quality over Quantity: Organic Compounds Altering the Antarctic Sea Spray Aerosol Concentrations

**DOI:** 10.1021/acs.est.5c07574

**Published:** 2026-01-05

**Authors:** Manuel Dall’osto, Matteo Rinaldi, Marta Estrada, Maria Dolors Vaqué Vidal, Elisa Berdalet, Ana Sotomayor, Miguel Cabrera-Brufau, Sebastian Zeppenfeld, David C. S. Beddows, Roy M. Harrison, Manuela Van Pinxteren, Hartmut Herrmann, Stefano Decesari, Marco Paglione

**Affiliations:** † Department of Marine Biology and Oceanography, Institute of Marine Sciences (CSIC), Pg. Marítim de la Barceloneta, 37-49, E-08003 Barcelona, Catalonia, Spain; ‡ Italian National Research Council—Institute of Atmospheric Sciences and Climate (CNR-ISAC), 40129 Bologna, Italy; § Atmospheric Chemistry Department (ACD), Leibniz-Institute for Tropospheric Research (TROPOS), D-04318 Leipzig, Germany; ∥ National Centre for Atmospheric Science Division of Environmental Health & Risk Management School of Geography, 1724 Earth & Environmental Sciences University of Birmingham, Edgbaston, Birmingham B15 2TT, U.K.

**Keywords:** primary aerosol, Antarctic aerosol, sea spray, marine atmospheric biogeochemistry

## Abstract

The Antarctic coastal zones are among the most biologically productive areas on Earth. The effect of marine microbiota on the emissions of sea spray particles, a critical factor for global climate and clouds, remains an open and actively researched question. Here, by means of in situ ship-borne bubble-bursting SSA production experiments at multiple locations around the Antarctic Peninsula, we show a 2-fold variability in the 10–500 nm size-resolved SSA number concentrations. We observed that the organic chemical composition of seawater (SW) and surface microlayers (SML) clearly impacts SSA number concentrations. SW and SML samples with saccharides, proteins, and N-osmolytes were less efficient at emitting SSA compared with waters rich in biotic material originating from lipids, such as fatty acids and polyols. We found that the dissolved organic carbon (DOC) fraction containing lipid degradation products and polyols indicates higher SSA production. Our results indicate that low concentration organic components, rather than the most abundant classes of biomolecules, influence the ability to be aerosolized, with strong chemical selectivity affecting SSA production.

## Graphical abstract

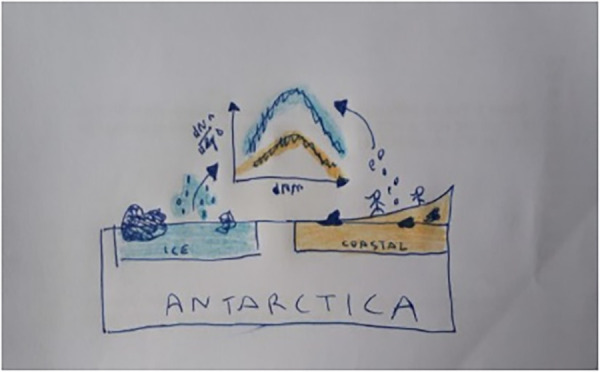

## 1 Introduction

The pristine region of the Southern Ocean (SO) and Subantarctic waters play a major role in modulating Earth’s climate.^1^ The annual freezing and subsequent thawing of the Southern Ocean surface close to the Antarctic continent establishes a vast expanse of ice. This, in turn, supports a massive and stable ecosystem for a wide range of microbial life.^2,3^ The network of environmental systems in this area is complex, and it includes open ocean currents, sea ice, and terrestrial snow and land cover. Such a network is showing clear signs of cryospheric and biological feedback mechanisms.^4–8^ Currently, climate models have large biases^9^ and the feedbacks between atmospheric and oceanographic biogeochemical processes remain poorly represented in Earth-system models (ESMs). In particular, aerosol sources and processes linking marine biota, marine aerosols, and cloud droplet number concentrations have still not been fully characterized. There is ongoing scientific debate regarding whether secondary aerosols—mainly generated from the atmospheric breakdown of dimethylsulfide (DMS), a gas released by marine plankton—or primary sea-spray aerosols play the more significant role in controlling cloud cover above the SO.^10–17^ SSA are produced when waves and splashes disrupt the surface, a process that is also active in sea ice regions like the edges of ice, ice floes within open pack ice, and consolidated marginal ice areas.^18^ Beyond the bulk surface seawater (SW), the sea surface microlayer (SML)–defined as a gelatinous film on top of the ocean^19^—also contributes to the sea-air transfer. McCoy et al.^13^ suggested that primary marine organic aerosols are important in this region. However, despite the increasing awareness of their importance, there are very few measurements of the composition and contribution to cloud condensation nuclei (CCN) of organic mass (OM) at different sizes for particles over the Southern Ocean.^16^ Saliba et al.^20^ found that the large organic fraction of particles <0.1 μm diameter may have important implications for CCN number concentrations and indirect radiative forcing over the SO. Recently, Humphries et al.,^21^ identified three main aerosol sources in the SO: the Northern (40–45 S), Midlatitude (45–65 S), and Southern sectors (65–70 S), with different mixtures of continental and anthropogenic, primary and secondary aerosols depending on the studied region. During the same period of study, Sanchez et al.^22^ found a weak gradient in CCN at 0.3% supersaturation with increasing CCN concentrations to the south between 44 and 62.1 °S, which may be caused by aerosol precursors from Antarctic coastal biological emissions.

Several studies highlight that the complex dynamics involving aerosols, clouds, and rainfall in the polar regions (higher latitudes) of the SO are markedly distinct compared to those occurring in the ocean’s midlatitude or lower latitude regions.^23,24^ However, the simple latitude gradient may not itself completely describe the aerosol variations observed. Indeed, recent literature shows that within the polar Antarctic air masses (>60° S) aerosol populations of very different chemical composition exist.^17^

Building on our previous findings that sea ice and associated ocean microbiota release primary and secondary organic nitrogen (ON) compounds (specifically low-molecular weight alkylamines), these ON compounds are crucial considerations when evaluating secondary aerosol formation processes in the Antarctic.^25–29^ Subsequent research suggests that the influence of the sea ice (sympagic) planktonic ecosystem on aerosol composition was previously underestimated, with several distinct eco-regions around Antarctica serving as unique aerosol sources.^28,30,31^ Additional complexity may come from insular terrestrial biomass emissions containing large amounts of OC.^32–34^ In Antarctica, the coastline extends for 17,968 km and comprises about 34.8–36.4 × 10^6^ km^2^, where 80% of this surface is covered by ice, even in summer.^35,36^ Overall, there are about 400,000 km^2^ of free ice coast shallower than 200m.^36^ The coastal regions of Antarctica support a rich array of marine and terrestrial life, notably including Antarctic seaweeds (benthic macroalgae) and populations of seabirds (primarily penguins).

The Antarctic Peninsula (AP) is affected by the greatest warming occurring in the Southern Ocean. Indeed, observations at the AP show pronounced shifts and reorganizations in regional ecosystems and biogeochemical cycles.^37,38^

To gain insight into the influence of marine Antarctic biogeochemistry on atmospheric aerosol, we report simultaneous water–air measurements made by means of in situ bubble-bursting SSA production experiments. In this study, our objective is to reduce the critical knowledge gap in our understanding of how the atmospheric aerosol chemistry of SSA is affected by the ocean biogeochemistry. SSA composition is expected to be altered by different surface seawaters with different biogeochemical properties.

Dall’Osto et al.^26^ were the first, to our knowledge, to present SSA production experiments using Antarctic melted sea ice and the bubble-bursting method, concluding that SSA aerosol concentrations are better correlated with the decay and maturity of the algal assemblage, rather than simply its biomass or production rate.

Here, we collected seawater samples from the open ocean as well as from the coastal systems around the Northern Antarctica Peninsula, which were placed in a bubbling tank designed to produce sea spray.^39,40^ The novel detailed characterization of different bubbled seawaters from Antarctic waters allows us to significantly improve our knowledge of Antarctic SSA and the connection to ecological and biogeochemical parameters influencing SSA production in an area of the SO where very few measurements exist.

## 2 Methods

### 2.1 Materials and Methods

#### 2.1.1 Bubble-Bursting SSA Production Experiments; Setup and Design

During the PI-ICE (Polar Interactions: Impact on Climate and Ecology) study conducted in the Northern Antarctic Peninsula (Figure S1) in January and February 2019, we collected a number of environmental samples including sea ice and water.^39,40^ The sampling sites are distributed between 67.8 °S in the western Peninsula, close to the Avian Island, up to 62.6 °S (Antarctic Johnson Bay in Livingston Island). To minimize contamination from ship exhausts and human contamination, water samples were taken at distances greater than 100 m from the ship or nearby coastal systems. The seawater (SW) samples were collected using a rubber boat. The SML samples were also obtained using a rubber boat, by immersing a glass plate (length: 50 cm, width: 20 cm, thickness: 0.5 cm) vertically into the surface water and slowly withdrawing it.^41,42^ Hence, the SML film was collected off the glass plate surface into a clean plastic bottle with a framed Teflon wiper. For most samples, we collected both the bulk water (hereafter SW) and the relative SML sample for biogeochemical analysis. Additionally, we collected about 25 L of SW, and we used it for the bubble-bursting SSA production experiments. Such experiments were performed on a 75 L airtight high-grade stainless-steel cylindrical tank illustrated elsewhere^39,40^ (see also Supporting Information—SI—and published information from our prior Arctic deployments of this system^43,44^ ).

In this study, we focus on a total of 9 Antarctic samples shown in Table S1. The factor analysis (Principal Component Analysis, PCA; non-negative factor analysis techniques, multivariate curve resolution, MCR; and Positive Matrix Factorization, PMF; see Section 2) is instead carried out on a larger number of variables (see Supporting Text S4–S5 and Table S2) to better separate the individual factor components. The reason for the selection of the 9 Antarctic samples is that they are the ones on which an almost complete set of characterization measurements were carried out (including both SW and SML and the SSA PM_1_ resulting from the bubble-bursting experiments and SMPS SSA size distribution measurements).

The biological organic matter produces surface-active agents that profoundly modify the water’s surface tension, viscosity, and density, thereby impacting the mechanisms of bubble-bursting and SSA release, which remain areas of limited scientific knowledge.^45,46^ It is important to acknowledge the constraint that ocean reality cannot be duplicated in the laboratory, yet these experiments are key tools for investigating and clarifying the basic mechanisms that drive ocean-atmosphere processes. In other words, a limitation of this study is that our laboratory experiments are representative of SSA produced in these different types of water using the specific experimental system that we are considering (a plunging jet aerosol chamber). Future studies should aim at developing specific polar aerosol chambers able to mimic the real-world conditions, for example, entrained bubbles in the open leads, or the interaction of smaller waves within a lead with solid sea ice material.

#### 2.1.2 Biological and Chemical Parameters Estimated in Seawater and SML Samples

Samples for the characterization of the biological and chemical properties in the seawater were taken before the 24-h-long bubble-bursting SSA production experiments. The water samples were consistently taken from the highest point in the marine aerosol chamber for convenience and to prevent any potential deposition of material at the chamber’s base. Details on the methodology and an extended description of the biogeochemical and microbiota components can be found elsewhere^39,40,47^ and in the Supporting Information. Briefly, seawater samples collected before or after the aerosol generation period were kept frozen until the analyses conducted at the laboratories within six months after collection for the following parameters: abundances and biomasses of virus (VA), prokaryote (PA), phototrophic (PNF) and heterotrophic (HNF) nanoflagellate and identification of phytoplanktonic communities; concentration of dissolved organic carbon (DOC), particulate organic carbon (POC), fluorescent dissolved organic matter (F-DOM), transparent exopolymer (TEP), Coomassie stainable particles (CSP), particulate and dissolved carbohydrates in seawater,^47^ Dissolved Fluorescent Organic Matter,^48^ and particulate water-extractable organic composition by proton nuclear magnetic resonance (1H-NMR) spectroscopy.^30^ All of the methods used are reported in the Supporting Information (Text S2). Dissolved organic matter (DOM) is an operationally defined complex mixture of organic molecules that passes through a submicron filter, while particulate organic carbon (POC) is combustible, noncarbonate carbon collected on a filter, typically >0.7 μm.^45^

#### 2.1.3 Bubble-Bursting Aerosol Chemical Characterization

The PM_1_ filter samples (see Supporting Text S1) collected during the bubble-bursting SSA production experiments were extracted in deionized water and analyzed by ^1^H NMR spectroscopy at 600 MHz using the same procedure employed for the SW and SML POC samples^30^ (SI Text S2). To reduce the complexity of the full chemical and biological data sets and to facilitate comparison of SW, SML, and SSA sample features, we applied a series of multivariate statistical techniques (see SI Text S3–S4). In summary, in this study, we characterize by ^1^H NMR the seawater (SW), the surface microlayer (SML), and the sea spray aerosol (SSA) samples. It is important to stress the difference between the SW-SML NMR sample set and the SSA NMR sample set analyzed by ^1^H NMR. While the former characterizes the water-extractable POC component, the latter has both the DOC and POC components. This is due to the technical limitation of not being able to characterize the DOC component in the SW-SML samples.^49,50^

## 3 Results and Discussion

### 3.1 SSA Production from Antarctic Waters

The size-resolved concentrations of the generated aerosols in the nine experiments were measured at 15 ± 10% relative humidity and are reported in Figure 1 (d*N*/d log dp, where dp is particle mobility diameter and concentration in cm^–3^). The average mode of the probability density function fitted to the particle number distribution is broadly seen at about 150–200 nm for SSA generated using breaking waves, consistent with previous marine pelagic studies,^51,52^ and other sea ice influenced (sympagic) air-sea aerosol transfer studies.^39,40^

**Figure 1:**
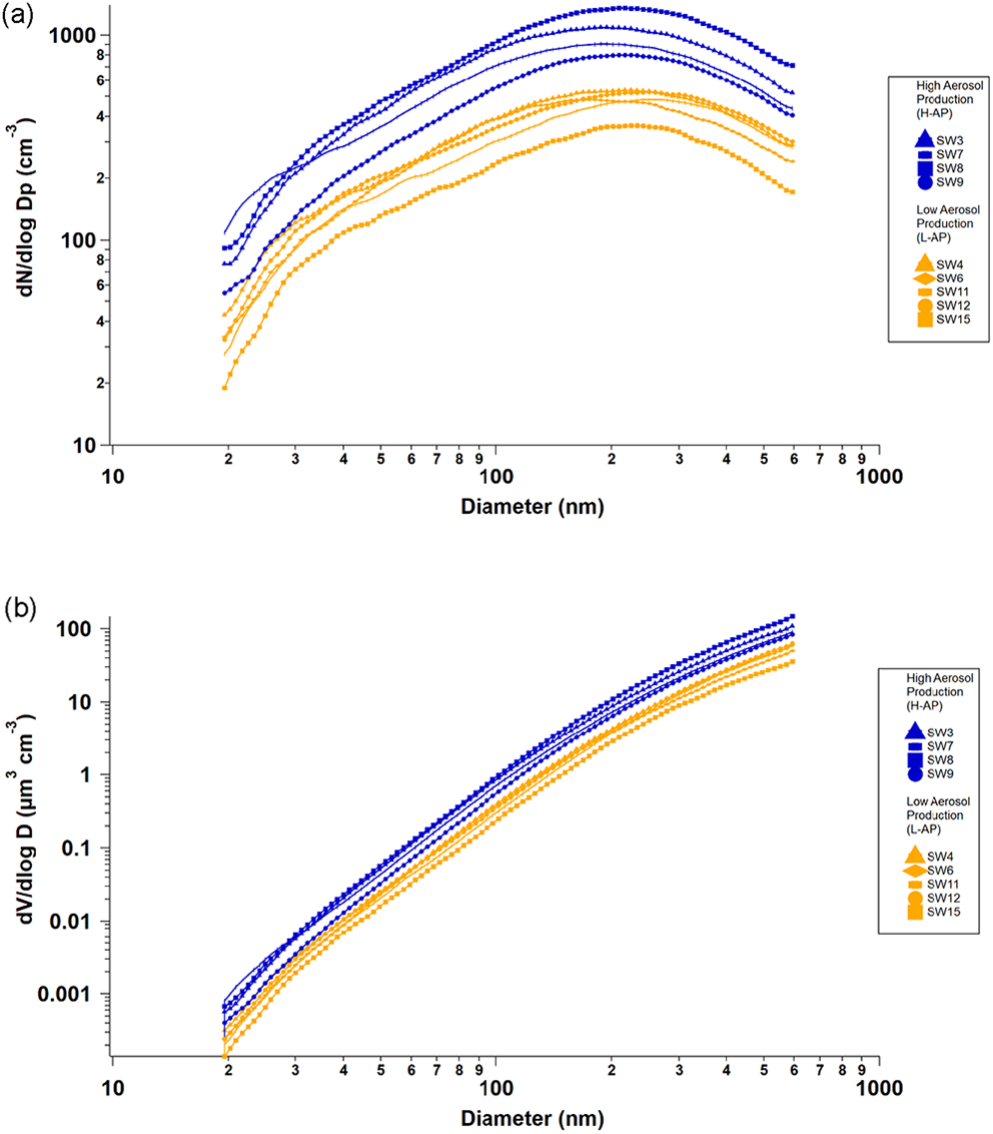
Aerosol size-resolved particle number (a) and volume (b) concentrations for the nine bubble-bursting SSA production experiments as L-AP and H-AP (low and high SSA production).

The plot of the nine Particle Size Distributions (PSD) shown in Figure 1 broadly shows two groups: PSD was characterized by low (L) and high (H) aerosol production (AP) loadings (L-AP and H-AP, respectively). Hence, following the visual classification, we colored five PSD (SW4, SW6, SW11, SW12, SW15) characterized by L-AP in dark orange and four PSD (SW3, SW7, SW8, SW9) characterized by H-AP in blue. The average particle number concentrations of the H-AP group (blue markers) are about a factor of 2 (210%) higher relative to the L-AP group (dark orange markers, Figure 1a). All distributions show only a small contribution from particles in the smaller Aitken mode (sub–100 nm size range).^51^ We fitted log-normal distributions for the size-resolved number concentrations (Figure 1a), and the results are shown in Supporting Figure S8. When the two aerosol size distribution groups are compared with each other, the average PSD in blue (*N* = 4, Figure 1) shows a bimodal distribution peaking at 35 ± 5 and 199 ± 10 nm. By contrast, the PSD in dark orange (*N* = 5, Figure 1) shows a trimodal distribution at 36 ± 4, 123 ± 10, and 280 ± 15 nm (see Figure S8 for details). The size-resolved volume concentrations (Figure 1b) show similar distributions among the two groups, peaking in the largest detected sizes, with the H-AP group having on average three times higher than the L-AP group.

### 3.2 Linking Ocean-Atmospheric Marine Chemical Properties

When looking at the location (Table S1 and Figure S1) of the nine samples discussed in Figure 1, most of the H-AP samples include all locations near marginal sea ice regions, whereas most of the L-AP samples originate from coastal sites. In particular, SW7, SW8, and SW9 were collected in marginal sea ice zone areas, whereas SW11, SW12, and SW15 were from the Antarctic Johnson Bay (at Livingston Island). SW4 and SW6 were collected close to coastal areas and near penguin colonies and benthic algae colonies. While temperature and salinity averages were not statistically different for the two L-AP and H-AP groups, the Secchi disk measurements for water transparency (Table S1) showed an average of 4.5 ± 2m for the L-AP relative to the 7.7 ± 1m for the H-AP group, somehow indicating waters with low turbidity associated with the H-AP group. Indeed, the water samples from the L-AP group exhibit higher DOC and substantially higher POC concentrations than the H-AP group (Table S4). Organic microgels (TEP and CSP) exhibit much higher concentrations in the conditions of L-AP than in the H-AP cases; this was unexpected based on the evidence of the ability of these classes of biological materials to enrich in the SML and in the sea-spray.^53–56^

#### 3.2.1 NMR Characterization of POC in SW-SML Water Samples and Links to Atmospheric Aerosol SSA Composition

##### 3.2.1.1 Principal Component Analysis Approach

The ^1^H NMR analysis was performed on the water extracts of all of the available POC samples from SW and SML (collected in the field) as well as of the SSA PM_1_ samples (collected from the aerosol chamber run in the field). For details, see Supporting Information Texts S3–S4 and Table S4. To reduce the number of variables, we applied a Principal Component Analysis (PCA) analysis to the whole set of NMR spectra recorded on both POC seawater samples (SW, SML) and corresponding SSA PM_1_ aerosol samples generated during the corresponding bubble-bursting SSA production experiments (NMR PCA results scores in Figure 2, NMR PCA loadings in Figure S2, and methods in Supp. Info Text S4). The two ensembles of water (POC from SW and SML) and air (PM_1_ SSA) samples show a clear split (square and circle group in Figure 2) in the PC1 dimension of the PCA (Scores in Figure 2, Loadings in Figure S2), indicating that the chemical composition of the water and the corresponding bubble-bursting SSA produced is remarkably different. Our findings confirm that the process of bubble-bursting acts in a highly selective manner.^42,57,58^ The loading plot of PC1 (Figure S2a) shows that N-osmolytes such as betaine, choline, and acetyl-choline (positive loadings)—so common in POC extracts on the right part of the scores diagram in Figure 2a—are transferred to a very limited extent to SSA (represented by negative loadings and left part of the scores diagram). Conversely, low-molecular-weight fatty acids or other aliphatic compounds of similar structure (represented by the negative PC1 loadings, Figure S2a) are prevalently found in the SSA and not in POC (left side of Figure 2a).

**Figure 2:**
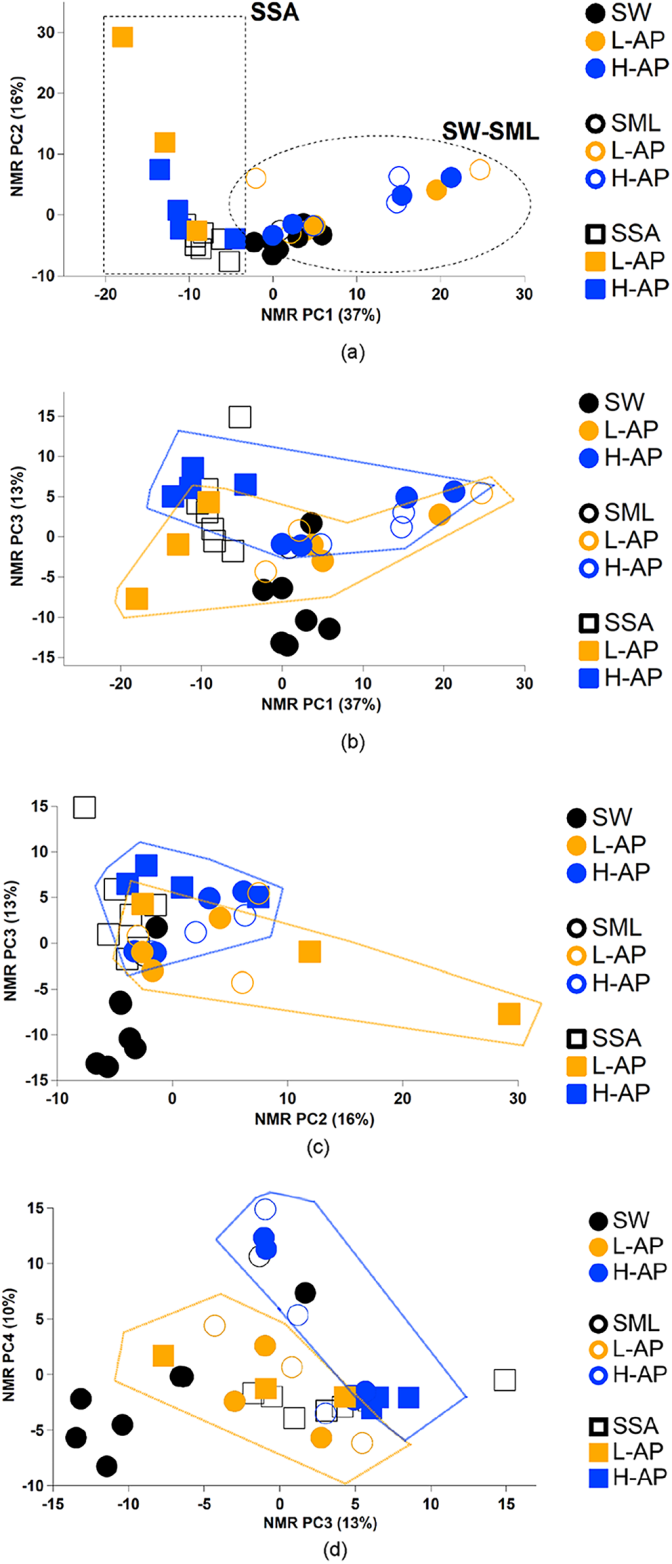
PCA scores plots for NMR spectra analysis of PC1, PC2, PC3 and PC4 as (a) PC1 vs PC2, (b) PC1 vs PC3, (c) PC2 vs PC3, and (d) PC3 vs PC4. Please note: Solid dots markers SW (Sea Water), empty dots markers SML (Surface Micro Layer), and square markers SSA (Sea Spray Aerosols). Dark orange markers are the L-AP group (*N* = 5), Blue markers are the H-AP group (*N* = 4), and the black markers are additional points where the complete sets of measurements are missing (either SW, SML, or SSA samples missing, see Tables S1 and S2).

When the SSA is produced, both the POC and DOC components are aerosolized, including the DOC compounds originating from POC via exudation or cell lysis. Given that we only characterized a fraction of the POC component of the SW and SML water samples (and not the Total Organic Carbon—TOC), the source of such aliphatic compounds (negative values of PCA PC1 both in Figures 2a and S2a) found in the aerosolized PM_1_ samples is likely in the Dissolved Organic Carbon (DOC) fraction of the water, herein tentatively attributed to the products of hydrolysis, oxidation or metabolism of lipids.

PC2 (Figure S2b) is associated with the variability in the content of lactic acid, a major product of sugars fermentation,^59^ which is found in seawater samples—SW and SML—collected at Adelaide Island (SW3, 4) and Davis Coast (SW8) but is especially representative of SSA samples resulting from SWs collected at Livingston Island (SW11, SW15).

A clear separation of the L-AP and H-AP groups is seen instead when the PC1 and PC2 components are plotted versus the PC3 component, as shown in Figure 2b,c, respectively. PC3 shows a clear tendency of the samples associated with high bubble-bursting particle number concentrations to spread at medium-high positive values: the higher positive scores for PC3 are all found for H-AP samples of bubble-bursting aerosol (SW 3, 7, 8, 9) and bulk seawater SW3, 8. Conversely, the lowest values are found for the SSA samples of SW11 and the seawater samples (bulk SW and SML) of SW15, which correspond to bubble-bursting experiments producing particles in relatively small numbers (L-AP). The loading plot of PC3 (Figure S2c) indicates that the H NMR spectral features of amino acids and saccharides are clearly characteristic of negative values of PC3, therefore, the enrichment of sugars and proteins in SSA is found to be associated with water, with little efficiency of generating SSA (L-AP group, Figure 1). By contrast, the positive loadings of the PC3 are associated with glycerol (Gly) and LMW fatty acids (and such compounds are associated with the H-AP group, top level of Figure 2b,c). Glycerol is renowned for its function in alleviating environmental stresses, such as those caused by freezing and hyperosmotic (high salt/sugar) environments. Antarctic extremophiles can produce glycerol as an osmoprotectant (and possibly a cryoprotectant^60^). Our study fully supports that of Decesari et al.,^30^ where glycerol was already previously associated with primary marine particles, in particular, glycerol accounted for almost the entire polyol content in sympagic water and aerosol samples, while the ones from the open pelagic ocean contained much larger and more complex mixtures of polyols/sugars.^30^

A final aspect of our PCA analysis can be described by the fourth component (PC4, Figure S2d), with positive signals of acrylic acid (acrylic acid, a degradation product of dimethylsulfoniopropionate, DMSP^61^), glycerol, acetic acid, lactic acid, iso-butyric acid, while being depleted of complex aliphatic compounds including both amino acids and lipids. The diagram of PC3 versus PC4 shown in Figure 2d confirms the separation of the two L-AP and H-AP groups seen in Figure 2b,c, with PC3 driving the difference. PC4 adds some insight into the complexity of the H-AP group, separating the SW-SML samples (circle markers) in the positive part of PC4 and leaving the SSA samples (square markers) in the negative part of PC4. This suggests that the bubble-bursting transfer of the H-AP group is far more selective than that of the L-AP group. The low-molecular-weight compounds found in the positive PC4 dimension trace the precursors of SSA aerosol in the water under H-AP condition (solid blue circles), but the same compounds are not found in the actual SSA produced, leaving the solid blue square (H-AP SSA) at the bottom of the chart in the negative side of PC4 (Figure 2d). Our PCA analysis of the NMR samples clearly shows that the organic components of the water samples are crucial in regulating SSA production, as we provide distinct “chemical fingerprints” for the H-AP and L-AP regimes.

##### 3.2.1.2 Chemical Quantification of SW-SML Samples by Non-Negative Factor Analysis Techniques Approach

The statistical results of the PCA reported in Figure 2 (and Figure S2) help us in identifying differences between seawater (SW and SML collected in the field) and aerosol samples (SSA from the aerosol chamber), including main differences among them. In a further analysis, we also applied non-negative factor analysis techniques Multivariate Curve Resolution—MCR and Positive Matrix Factorization (PMF; see SI) which allow constraining the non-negativity of spectral profiles and contributions of the samples, therefore to quantify them and make their interpretation more physically meaningful. Because of the different nature of the dataset (water and atmospheric aerosols), the data sets of the SW-SML NMR spectra and SSA NMR spectra were analyzed separately, and detailed results are shown in Figures S3 and S4, respectively.

Regarding the POC of the SW-SML dataset, a five-factor solution obtained by the agreement of the PMF and the MCR-ALS analysis was found to best represent the separation of the interpretable spectral features (Figure S3). Important external correlations strongly support our Factor Analysis solution (Table S5), and the five factors found in the SW-SML samples can be summarized as

(1) SW-SML Factor 1 “saccharides”, composed of a large variety of compounds including glucose and sucrose. The abundance of Factor 1 correlates with the concentrations of both particulate and dissolved sugars speciated by LC/MS (Table S4).
(2) SW-SML Factor 2 “N-osmolytes”, composed of Nitrogen-containing osmolytes (Bet: betaine; Cho: choline and Acetyl-choline) typical of biological exudates, and with aminosugars (Table S4).
(3) SW-SML Factor 3 “proteins”, composed of amino acids such as alanine, leucine, isoleucine, tyrosine, threonine, phenylalanine; correlating with fluorescent DOC protein-like components (peaks B and T) and TEP and CSP (Table S5).
(4) SW-SML Factor 4 “lipids/polyols A” composed of a complex mixture of unidentified aliphatic organic matter, including signature low-molecular-weight fatty acids and acetate.
(5) SW-SML Factor 5 “lipids/polyols B” composed of aliphatic compounds such as low-molecular-weight fatty acids, and including signature acrylic acid and glycerol.

The SW-SML factors 1, 2, and 3 are associated with constituent biomolecules (proteins, polysaccharides), osmolytes, and exudate materials (TEP), while factors 4 and 5 represent something different, including degradation products (acrylic acid) and products of metabolism (lactic acid). Most of the indicators of productivity, DOC and POC correlate with the first three factors, but not with Factors 4 and 5. Beside the strong correlations explained above, the weak correlation of the fluorescent DOM “biological index” (i.e., the ratio of emission intensity at 380 nm divided by 430 nm at excitation 310 nm^62^) with Factor 4 (*r* = 0.21) is comparable to that with Factors 1 to 3 (*r* = 0.17–0.18) and larger than with Factor 5 (*r* = −0.02), indicating that Factor 4 is still more “freshly produced” than Factor 5 (Table S5). Interestingly, Factors 1 to 3 are weakly negatively correlated (*r* = −0.29/-0.35) with the fluorescent DOM “humification index”,^63^ and positively correlated with Factors 4 and 5 (*r* = 0.21/0.05) suggesting that humic substances do not contribute to Factors 1 to 3 but could contribute to a variable extent to Factors 4 and 5, although the correlations are weak. In a nutshell, Factors 1–3 are associated with saccharides, proteins, and N-osmolites, whereas Factors 4–5 are associated with lipids, polyols, and fresh humic material.

We looked for any link between the POC and DOC collected and the two groups of samples generating H-AP and L-AP concentrations (Table S4 and Figure 3, bottom). Both the total dissolved and the particulate organic carbon components (DOC, POC) of the waters used for aerosol generation did not have statistically different concentrations. However, we found statistically significant differences for polysaccharidic transparent exopolymer particles (TEP) and proteinaceous Coomassie stainable particles (CSP)—a fraction of exopolymeric substances (EPS) representing colloidal polymeric organic substances outside the cell.^64^ For this specific part of the POC measured, the L-AP samples had TEP and CSP values higher than those of the H-AP samples (Figure 3 and Table S4). As highlighted above, this is in contrast to previous studies, where a correlation was found between TEP and particle number concentrations.^26,55,56,65^ Figure 3 (middle) shows the chemical composition of the SW and relative SML of the bubble-bursting SSA production experiments shown in Figure 1. It is clear that all samples are enriched in organic mass in the SML relative to the corresponding SW sample. Additionally, the factor that is enriched the most in the SML fraction relative to the SW sample is found to be Lipids/polyols A (acetic, acrylic acid). This is also the component that is found in the highest amount in the H-AP group, somehow suggesting that this component enhances the aerosol production. By contrast, higher concentrations of saccharides are found in the L-AP group, somehow lowering the aerosol production efficiency. Our results are in line with independent laboratory experiments recently published by Xu et al.,^66^ further expanding them for the first time to data from real ambient waters.

**Figure 3:**
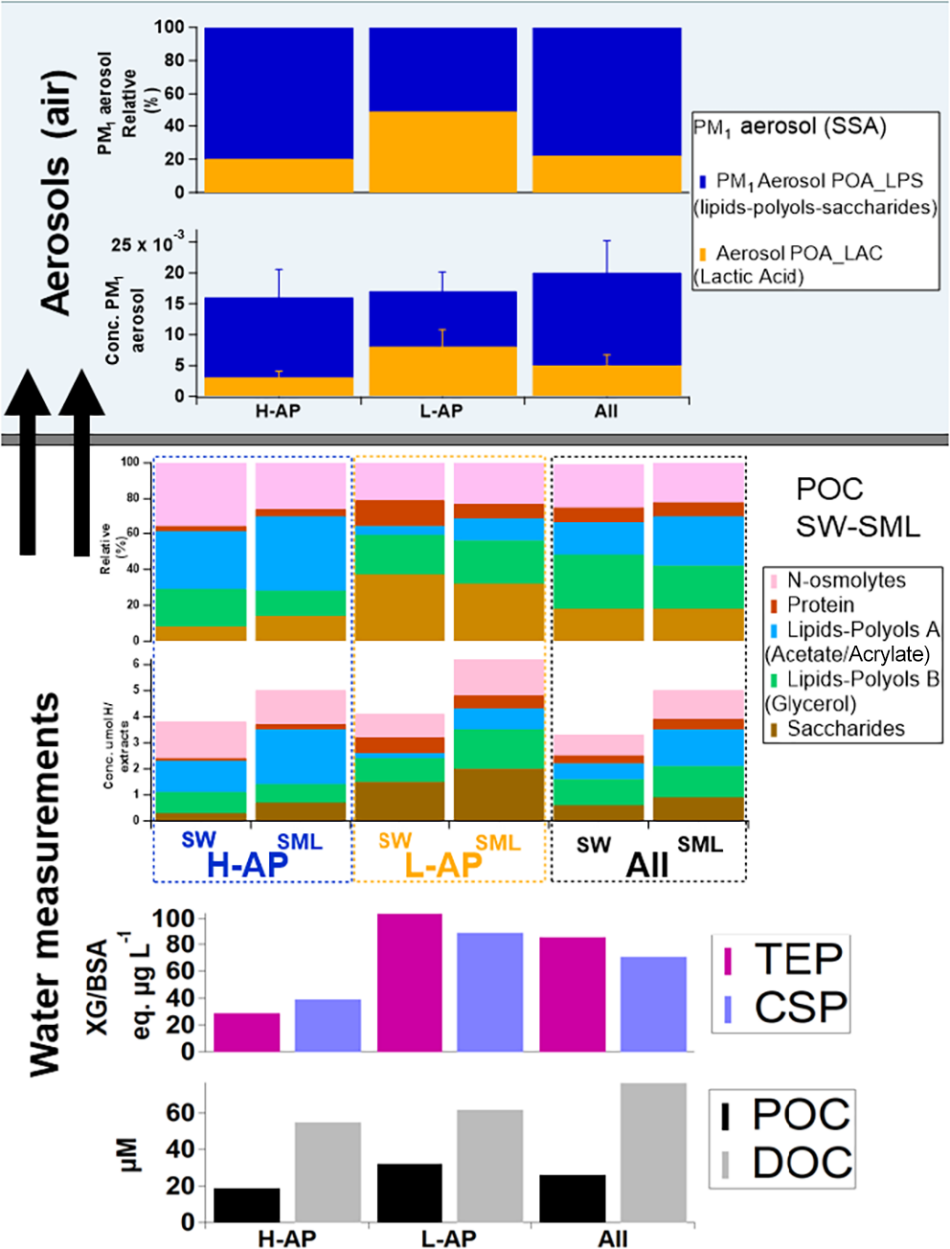
(Bottom panel) averages for dissolved organic carbon (DOC) and particulate organic carbon (POC); polysaccharidic transparent exopolymer particles (TEP) and proteinaceous Coomassie stainable particles (CSP), and (middle panel) PMF factor contributions for Sea Water (SW) and Surface Microlayer (SML) samples for the H-AP (*N* = 4), L-AP (*N* = 4) and All (*N* = 21) POC groups, and (top panel) PMF factor contributions for the H-AP (*N* = 4), L-AP (*N* = 3), and all (*N* = 8) bubble aerosol PM_1_ groups.

##### 3.2.1.3 Chemical Quantification of SSA Samples by Non-Negative Factor Analysis Techniques Approach

We applied the non-negative factor analysis also on the available PM_1_ SSA samples collected during the bubble-bursting SSA production experiments of PI-ICE^39,40^ (Tables S1 and S2 and Supporting Texts S3–S4). A broad two-factor solution was found to describe the Primary Organic Aerosol (POA) in the PM_1_ fraction, as shown in Supporting Figure S4 and in Figure 3 (top). The first factor (SSA_Factor 1–Lipids-polyols-saccharides) is characterized by aliphatic chains with terminal methyl moieties typical of lipids (bands at 0.9 and 1.3 ppm) associated with polyols (e.g., glycerol, among others, in the region 3.2–4.0 ppm). The occurrence of saccharides among polyols cannot be ruled out, and the resonance patterns found in POC Factor 1 are not recovered here. The second Factor (SSA_Factor 2–Lactic Acid) instead has sparse signals of a different mixture of aliphatic chains and polyols (including glycerol signals again) and is mainly characterized by the strong contribution of lactic acid. Noticeably, the organic components characteristic of POC Factors 1, 2, and 3 (saccharides, proteins, and N-osmolites) are found only in trace amounts in the SSA samples if not absent.

##### 3.2.1.4 On the Ocean-Atmospheric Marine Chemical Properties

Two important conclusions can be drawn from all of the ocean-atmospheric data shown in Figure 3. First, we provide evidence that different types of sea-spray POA exist. Second, while a lipid-polyol-saccharides component (similar to that found in previous studies^30,53^ ) is found associated with H-AP samples, a lactic acid—enriched component seems to be associated with L-AP samples (Figure S5). Moreover, Paglione et al.^28^ found the same factors representing POA components in ambient PM_1_ samples collected at two Antarctic stations around the Weddell Sea, namely Signy and Halley, demonstrating the ambient relevance of these components on the aerosol burden of a wide area. It is important to stress that the lipid-polyol-saccharides component found in the PM_1_ SSA aerosol phase can originate from both DOC and POC components of SW and SML. Although SSA-Factor 2 shows resemblance with SW-SML Factor 4 in the NMR spectral features, SSA-Factor 1, with its intense lipid resonances, has no equivalent in the characterized POC in SW and SML. This points to sources in distinct pools of organic material in SW, hence in the most surface-active fraction of DOC. Our biogeochemical results suggest that fresh organic material - possibly dissolved organic carbon (DOC) from lipids, polyols, and possibly humic-like substances - influences aerosol production.^51,52^

### 3.3 On the Relationships between Aerosol Production and Phytoplankton Composition

Understanding the spatial and temporal distribution of microbial components is essential to detect trends and predict their response upon sea spray production. Phytoplankton blooms in the Atlantic sector of the SO tend to be dominated by diatoms or haptophytes like *Phaeocystis* spp.^67^ Indeed, in previous studies in the same study area (Southeast of the South Orkney Islands^68^), the most important groups were haptophytes, followed by diatoms, Phaeocystis-like, and pelagophytes.^68^ The presence of cryptophyte communities has been associated with ice-melt-influenced, shallow mixed layers in several investigations; this change from a dominance of diatoms to cryptophytes is considered representative of the seasonal progression of phytoplankton in the West Antarctic Peninsula.^69^ The relationships between aerosol production and phytoplankton composition were explored by means of a PCA based on 21 selected taxa. The variance explained by the first three components of this analysis was, respectively, 22.7%, 21.2%, and 18.9% of the total (Figure S6). PC1 presented high positive loadings (or correlation coefficients) with large phototrophic nanoflagellates (PNF 5–20 μm), cryptopytes, *Gyrodinium* spp., and small unidentified dinoflagellates, while the small *Thalassiosira* spp. (average diameter <20 μm) showed the strongest negative loading (Figure S6). PC2 was positively correlated with medium-sized and large *Thalassiosira* spp, several pennate diatom taxa, and small and large unidentified dinoflagellates, and negatively correlated with *Corethron pennatum* and *Fragilariopsis* cylindrus (Figure S6). PC3 was positively associated with small PNF (2–5 μm) and Phaeocystis-like sp., while on the negative side, the strongest loadings were presented by the diatoms *Amphora* sp. One and *Fragilariopsis* spp. (Table S6).

The distribution of the scores of the samples in the space of these components is shown in Figure S7. As can be seen in the PC2 versus PC1 graph (Figure S7a), SW3 and SW4 (which were geographically close) occupy adjacent positions in spite of their differences in PSD (Figure 1). SW 7–8–9 (H-AP) presents low PC2 scores, whereas SW 4–11–12–15 (L-AP) shows an opposite trend. With respect to PC3, the highest scores were presented by L-AP samples SW12 and SW15 (Figure S7b,c). While a clear consistent ordination cannot be seen for all 9 points, high positive PC1 scores tend to be associated with high aerosol production, whereas high PC2 and PC3 scores correspond to low aerosol production. These results indicate that different biological communities, influencing the chemical composition of seawater, can lead to different efficiencies of SSA production/emission to the atmosphere. At the same time, it appears clear that the distributions of the main phytoplankton species, especially diatoms and haptophytes, which account for the largest taxonomic groups in this analysis, are influenced by several ecological factors which cannot be put in a net relationship with the two SSA production regimes H-AP and L-AP.

Nevertheless, when we contrast the distribution of phytoplankton with the concentrations of Chlorophyll, TEP and CSP and with its composition (Table S6), we can observe that the L-AP samples SW11, SW12 and SW15, with high positive scores on PC2 and PC3, corresponded to a peak of high Chl-a and phytoplankton biomass (mainly contributed by *Phaeocystis* sp. and diatoms, including various *Thalassiosira* spp. size fractions and *Pseudonitzschia* spp., Table S4), in line with high amounts of TEP and CSP. Different phytoplankton species produce EPS—including TEP and CSP—but they also change their secretions along with different stages of their life cycles, and in relation to various types of stress.^70^ Mucus secretion in relation to blooms of *Phaeocystis globosa* and its effect on seawater viscosity and surface foaming was already investigated in previous studies.^71–74^ Subsequently, a positive relationship between *Phaeocystis antarctica* and seawater viscosity in Antarctic waters was found.^75^ Other studies—although not taken in Antarctic regions and hence difficult to compare—also suggest a substantial impact of chlorophyll a on sea spray fluxes.^76–80^

In contrast, the high aerosol production samples presented lower average Chl-a values (although the difference was not significant; Table S4). SW7 and SW8, with relatively high scores on PC1, were populated by large phototrophic nanoflagellates (i.e., cryptophytes), confirming previous PI-ICE studies^39,40^ showing that some nanoflagellates are an important component in enhancing sea spray aerosol production. Table S4 also shows the microbial components measured (prokaryote, protist, viral, and nanoplankton abundances and biomass) for the two groups of bubble-bursting SSA production experiments characterized in Figure 1. None of the averages presented showed statistically significant differences, indicating that, also in terms of microbial composition, a clear difference between conditions responsible for the L-AP and H-AP regimes could not be found. Nevertheless, contrary to diatom abundance, chlorophyll concentration, the biochemical components and sugars, which on average all occur at higher concentrations in the L-AP sites, several of the microbial parameters, specifically the V1 and V2 viruses, the HDNA bacteria, and the phototrophic nanoflagellates (PNF) are higher at the H-AP locations.^39,40^

High chlorophyll concentrations and large numbers of nanoflagellates (especially *Phaeocystis*) are recognized as relatively consistent features of the Marginal Ice Zone (MIZ) and the northern waters influenced by the Antarctic Slope Front-Antarctic Slope Current system.^81^ Sellegri et al.^52^ also recently reported a consistent significant relationship between seawater nanophytoplankton cell abundances and sea-spray derived Cloud Condensation Nuclei (CCN) number fluxes. The high SSA production conditions observed during PI-ICE can therefore be put in relationship with the abundance of nanoflagellates relative to the other plankton communities (diatoms, dinoflagellates) because of the presence of sea ice or because of the greater buoyancy with respect to diatoms during the process of bubble bursting,^39,40^ in connection with the direct or indirect role of the same nanoflagellates in the production of DOM components which seems more efficient than TEP and CSP in producing high amounts of film drops and submicron sea spray particles.

## 4 Atmospheric Implications in an Antarctic Changing Climate

After the detailed chemical descriptions of the organic compounds identified in SW, SML, and SSA samples, here, we try to discuss broader scientific implications highlighted to bridge disciplinary boundaries.

### 4.1 New Insights from the Presented Dataset

Given that our main objective in this study is to understand the Sea Spray Aerosol (SSA) production of seawater (SW) from different marine Antarctic ecosystems, in this study, we categorized two main groups following the high and low aerosol number particle production.

The new insights gained from the presented dataset are

(a) Major SW-SML constituents of POC, like nitrogenated metabolites, saccharides, and proteins, represent only minor constituents of the submicron aerosol phase (PM_1_).
(b) Water samples rich in proteins occur in most eutrophic waters and are associated with low particle number production in spite of the large pool of organic material in the surface water available for aerosolization, meaning that the actual chemical composition of the surface water has an impact on the formation of film drops in this environment.
(c) The SSA high aerosol production regime is associated with surface water components other than saccharides, proteins and metabolites and more closely with products of degradation of lipids and polysaccharides.
(d) The measured SSA chemical composition of L-AP is characterized by organic matter with high contributions of lactic acid and a few polyols (such as glycerol). By contrast, H-AP shows low-molecular-weight fatty acid chains (possibly combined in larger, more complex chemical structures) associated with polyols. Coastal material is potentially associated with lower aerosol production efficiency, whereas cleaner (lower turbidity measured by Secchi Disk) waters with higher abundance of nanoflagellates (possibly from sea ice melting) enhance aerosol production efficiency. We suggest that it is not the amount of dissolved organic material in the SW-SML that matters but its chemical composition that strongly regulates the SSA production.

Understanding the role of organic chemical composition in determining the rheological properties of the SML relevant for particle generation by bubble bursting, such as surface tension, viscoelasticity of the film, viscosity and density of the fluid, film thickness and rupture thickness^82–85^ deserves further investigation, because of the complexity of the interactions in the mixture, as well as between ions and organic molecules.^86^ Studies such as^87^ highlight that a range of molecular organizations and film structures can originate from the mixture of a still limited set of fatty acids, proteins, and sugar compounds. Expanding similar approaches to compound mixtures including components of microbial DOM (such as fresh humic substances) would nicely complement the current experimental efforts aiming to determine SSA-forming properties of the marine SML in real-world conditions n^83^.

### 4.2 Specific Contribution to the Interdisciplinary Research in the Antarctic Peninsula

Our findings highlight the importance of distinct Antarctic SSA emissions and call for further studies of ocean–atmosphere relationships in the Antarctic Peninsula.

The present study points to start revealing underlying mechanisms involved in ocean–atmosphere processes, including a better understanding of how the biogeochemical properties of the Antarctic surrounding waters are controlling the SSA production, in areas of open water, leads, melt ponds as well as from other bubble formation processes.^88–91^

The work by Brean et al.^92^ highlighted the striking dissimilarities in aerosol particle size distributions throughout the Antarctic, which are likely a result of the region’s diverse eco-regions, where various atmospheric chemical and physical processes create several distinct aerosol sources. These findings^92^ indicate that the PNSD of small Antarctic aerosols (submicrometre) was likely overgeneralized in earlier research.^93^

The Antarctic is defined by complex bioregions that feature high spatial heterogeneity across its marine, terrestrial, and freshwater biomes, showing patchy biodiversity and productivity despite strong environmental gradients.^94,95^ This area is chiefly composed of coastal tundra, sea ice, and the ice-covered landmass, plus two marine regions: the Antarctic Peninsula and Scotia Sea and the Subantarctic Indian Ocean Islands.^96^ As the Antarctic environment transforms, it will induce climatic changes via interactions and feedback involving the biosphere and cryosphere. Specifically, adaptations in ecophysiology, food, and nutrient availability among marine and terrestrial organisms will directly impact the release of primary and secondary aerosol precursors.^92^

Our findings report data from coastal Antarctica and nearby open ocean (about 100 km from coastal systems), and further open ocean measurements across the Antarctic coastal system and the open Southern Ocean are advocated.

Burrows et al.^97^ introduced a novel framework for the parametrization of the organic fraction of SSA, modeling the contribution to SSA of mixtures of organic components with different properties (surface activity, molecular mass, and surface packing) in competitive equilibrium with each other. It was proposed that lipids contribute the majority of the SSA organics in the Southern Ocean regions of high productivity. Instead, in less productive waters, organic mass is primarily contributed by proteins, with polysaccharides providing a non-negligible contribution. Biogeochemically aged organic mixtures (recalcitrant surface DOC and abyssal humic-like substances) resulted in an overall negligible contribution. Our results are consistent with the above findings, showing the high potential for SSA transfer of dissolved organic carbon, including lipids and their freshly produced products of humification. Our results confirm the observations made by Gu et al.^98,99^ that show the summer carbonaceous aerosols above the Arctic Ocean are largely controlled by the marine fresh carbon pool,^98^ and that the Southern Ocean’s primary marine aerosol also contains a markedly greater contribution from this fresh carbon source than is seen at the Indian Ocean’s midlatitudes.^99^

Our biogeochemical results suggest that fresh organic material, possibly dissolved organic carbon (DOC) and Dissolved Organic Nitrogen (DON) from humic-like substances, influences aerosol production. We suggest that the freshly produced organic component produced by the microbial loop, a complex relationship in an ecosystem, is an important component of the ocean-atmosphere coupling, leading to an enrichment of SSA, eventually impacting the changing climate.

Furthermore, the results presented in this study not only confirm the lower capability of proteins and metabolites to be enriched in SSA with respect to lipids but also suggest that seawaters enriched in such biochemical components may produce a lower SSA particle flux than lipid-enriched waters under the same conditions. Predictions by Burrows et al.^97^ show that adsorption-driven enrichment of organic matter in SSA is correlated with chlorophyll concentrations in highly productive waters, in agreement with large-scale field observations. Leon-Marcos et al.^100^ also found that polar lipids contribute the most to the organic matter in aerosols, given the high air-seawater affinity of lipids compared to other groups. The present study suggests that ocean-atmospheric transfer mechanisms must be considered when comparing water samples with atmospheric aerosol samples, and interdisciplinary studies of this kind are essential to fully understand how marine biogeochemistry influences primary sea spray emissions in Antarctic regions, especially, given that it is the only natural preindustrial baseline left on our planet Earth.

The Antarctic region is characterized by a large spatial heterogeneity with marine, terrestrial, and freshwater biomes interacting with distinct environmental gradients. The quantification of global CCN concentrations is crucial for quantifying the radiative forcing. Significant inconsistencies currently exist in the data sets for pristine oceans, especially in the Southern Hemisphere, where the data not only indicate opposite seasonal shifts but also reveal contrasting annual patterns.^101^ Such discrepancies can reflect a too coarse representation of ecosystem-ocean-atmosphere interactions in an environment characterized by strong environmental contrasts, such as the Subantarctic oceanic areas and coastal Antarctic regions. The Antarctic Peninsula, in particular, is most vulnerable to climate change, with rapid ice shelf melting, coastal sea ice thinning and receding, retracting glaciers, and greening over land. Understanding how such dramatic environmental change will provide feedback on marine ecosystems and atmospheric emissions of CCN and reactive compounds represents a major scientific challenge. While opportunities can emerge from research on the analogy with the present-day climate in other polar areas,^102^ some studies have already highlighted changes in the biodiversity of phytoplankton consequent of the loss of sea-ice around the Antarctic Peninsula.^103^ On the basis of our findings, the loss of sympagic phytoplankton communities and the increase of coastal benthic communities in seabeds newly exposed by glacial retreat could result in a net change (potentially a decline) of the SSA formation potential. However, the complexity of the response of the microbial loop to climate change and its effects on marine biogeochemistry and ocean-to-atmosphere fluxes call for more research. Therefore, we suggest additional studies to quantify the connection between the changing Antarctic marine biogeochemistry and atmospheric composition, likely to involve cloud feedbacks that are yet poorly understood.

## Supplementary Material



## Data Availability

All data are available upon contacting the corresponding author. The dataset used in the manuscript can be found in the Zenodo repository at doi: 10.5281/zenodo.17338678.

## Notes

Study area and specific ocean-atmospheric tank methodology (Text S1); extensive methodology for biological and chemical parameters estimated in the seawater (Text S2); SW, SML, and sea-spray aerosol (SSA) chemical characterization by _1_H NMR (Text S3); factor analysis of H NMR POC & SSA spectra (Text S4); statistical analysis of the main phytoplankton communities (Text S5); sampling time and information for the selected samples (Table S1); short description of all the water samples collected (Table S3); H NMR identified/measured chemical species/categories (Table S3); marine biogeochemical variables (Table S4); correlation between categories of compounds identified by H NMR PMF analysis and the chemical/biological composition of the seawater/sea-ice samples (Table S5); coding for the 21 selected phytoplankton variables (Table S6); map of the study area (Figure S1); PCA loadings of the PCA H NMR analysis (Figure S2); PMF ME-2 and MCR-ALS profiles and contributions of the 5-factors solution from POC dataset of NMR spectra factor analysis (Figure S3); PMF ME-2 and MCR-ALS profiles and contributions of the 2-factors solution from SSA dataset of NMR spectra factor analysis (Figure S4); PMF results of the NMR PMF F1 (POA lipid-polyols-saccharides) and NMR PMF F2 (POA Lactic Acid) (Figure S5); PCA phytoplankton results (Figure S6); PCA phytoplankton plots (Figure S7); and PNSD presented in Figure 1a, but not in the Y log mode (Figure S8) (PDF)

## ⊥

Department of Environmental Sciences, Faculty of Meteorology, Environment and Arid Land Agriculture, King Abdulaziz University, Jeddah 21589, Saudi Arabia

## #

Departament de Biologia Evolutiva, Ecologia i Ciències Ambientals, Universitat de Barcelona, Av. Diagonal 643, 08028 Barcelona, Spain
