# Supplementary material for: Quality over Quantity: Organic Compounds Altering the Antarctic Sea Spray Aerosol Concentrations
Source: Environ Sci Technol. 2026 Jan 5;60(3):2490–502. doi: 10.1021/acs.est.5c07574 (PMC12854742; doi:10.1021/acs.est.5c07574)
Supplement: Supplementary file 1 [file es5c07574_si_001.pdf]

SUPPLEMENTARY INFORMATION

Quality over quantity: organic compounds altering the Antarctic Sea spray aerosol concentrations

Manuel Dall'Osto[1*&], Matteo Rinaldi[2], Marta Estrada[1], Maria Dolors Vaqué Vidal[1], Elisa Berdalet[1], Ana Sotomayor[1], Miguel Cabrera-Brufau[1†], Sebastian Zeppenfeld[3], David C.S. Beddows[4], Roy M. Harrison[4 ǂ], Manuela van Pinxteren[3], Hartmut Herrmann[3], Stefano Decesari[2] and Marco Paglione[2&]

[1]Department of Marine Biology and Oceanography, Institute of Marine Sciences (CSIC), Pg. Marítim de la Barceloneta, 37-49, E-08003 Barcelona, Catalonia, Spain;

[2]Italian National Research Council– Institute of Atmospheric Sciences and Climate (CNR-ISAC), 40129 Bologna, Italy

[3]Atmospheric Chemistry Department (ACD), Leibniz-Institute for Tropospheric Research (TROPOS), D-04318 Leipzig, Germany

[4]National Centre for Atmospheric Science Division of Environmental Health & Risk Management School of Geography, Earth & Environmental Sciences University of Birmingham, Edgbaston, Birmingham, B15 2TT, UK

[ǂ] Also at: Department of Environmental Sciences, Faculty of Meteorology, Environment and Arid Land Agriculture, King Abdulaziz University, Jeddah 21589, Saudi Arabia

† Current adrress: Departament de Biologia Evolutiva, Ecologia i Ciències Ambientals, Universitat de Barcelona, Av. Diagonal 643, 08028. Barcelona, Spain

[&] both equally contributed

*Correspondence to Manuel Dall´Osto : dallosto@icm.csic.es

Number SI pages consecutively starting with page S1: 27 pages

Number of Supplementary Text 5 (Text S1-S5)

Number of Tables 6 (Table S1-S6)

Number of Figures 8 (Figure S1-S8)

Text S1. Study Area and specific ocean-atmospheric tank methodology.

The sampling sites are distributed between 67.8 °S in the western Peninsula, close to the Avian Island, up to 62.6 °S (Antarctic Johnson Bay in Livingston Island). The laboratory sampling chamber "Olla" consisted of an airtight high-grade stainless-steel tank, details and schematic can be found elsewhere where it was already deployed in both Antarctic (Dall´Osto et al., 2022 a, b) and Arctic regions (Rocchi et al., 2024, Li et al., 2024). Briefly, the tank was filled only with water (25 L total volume for each bottle). The chamber was kept refrigerated keeping the chamber at a constant temperature of about 2±0.5º C. It is imperative to stress the limitation of our study, cause laboratory experiments cannot mimic the real conditions of the ocean, nevertheless they can help to unveil underlying mechanisms involved in ocean-atmosphere process. Water was cycled out from the bottom of the tank and injected back in at the top of the tank as a plunging jet at a rate of 10 L min^{-1} (Dall´Osto et al., 2022 a, b). The entrained jet formed bursting bubbles on the surface of the water, thus producing sea spray aerosol. Particle-free compressed air was swept into the tank headspace (60 L min^{-1}), which had outlet ports including a PM1 (Particulate Matter smaller than 1 µm) aerosol sampler (47 mm Whatman QM-A quartz filters) with air pumped at 38.3 L min^{-1} flow rate. PM_1 aerosols (PM impactors, TRC Tecora, Monza and Brianza, Italy) samplers were used for about 24h to collect enough material to be analyzed in the laboratory. Number size distributions of the aerosol, across the 10–500 nm size range, were collected using a Scanning Mobility Particle Sizer (SMPS; DMA TSI 3080 and CPC TSI 3025, corrected for diffusion losses, silica gel dryer used) with scan times of 5 minutes. Sampling locations and short description of each SW and SML sample collected during the PI-ICE project are reported in Fig. S1 and Tab. S1 and Tab. S2.

Text S2. Extensive Methodology for Biological and chemical parameters estimated in the seawater.

- Virus and prokaryote abundance Virus (VA) and prokaryote (PA) abundances were assessed by flowcytometry following the protocols of Boras et al. (2010) and Gasol and Del Giorgio (2000), respectively at the ICM-CSIC laboratory.

- Nanoflagellate abundance and biomass. Cell counts were conducted by epifluorescence microscopy, previously stainrd with DAPI (Sieracki et al. 1985). Phototrophic nanoflagellates (PNF) could be distinguished from heterotrophic nanoflagellates (HNF), based on red fluorescence emission by plastidic structures upon blue light excitation, (Dall´Osto et al., 2022a, b).

- Phytoplankton identification, abundance and biomass of lugol fixed samples were counted using an inverted XSB-1A microscope.

- Chlorophyll a. Concentrations were determined by fluorimetry with a calibrated Turner Designs fluorometer following the method developed by Holm-Hansen et al (1965).

- Dissolved Organic Carbon (DOC) Measurements were conducted with the TOC-V CSH Shimadzu autoanalyzer that holds a NDIR (non-dispersive infrared) detector.

- Particulate Organic Carbon (POC) measurements were made with an elemental analyser (Perkin-Elmer 2400 CHN).

- Fluorescent DOM determinations were carried out using a Horiba Aqualog spectrofluorometer. Excitation-emission matrices (EEMs) were collected in the excitation/emission range 240-600/240-600nm and the EEM spectra were analyzed according the procedure described in Dall'Osto et al. (2022a,b).

- Transparent Exopolymer (TEP) and Coomassie Stainable Particles (CSP) concentrations were determined following the colorimetric method described by Passow and Alldredge (1995) and Cisternas-Novoa et al. (2014), respectively.

-Particulate and Dissolved Carbohydrates in seawater. Particulate combined carbohydrates (PCCHO, >0.2µm), dissolved combined carbohydrates (DCCHO, <0.2µm) and dissolved free carbohydrates (DFCHO) were determined as described by (Zeppenfeld et al., 2020, 2021) using a high-performance anion-exchange chromatography with pulsed amperometric detection (HPAEC-PAD). DFCHO, DCCHO and PCCHO were calculated as the sums of following monosaccharides, either occurring as free molecules (DFCHO) or being released after an acid hydrolysis (DCCHO & PCCHO): glucose, fucose, arabinose,

rhamnose, galactose, xylose, mannose, fructose, glucosamine, galactosamine, muramic acid, glucuronic acid, galacturonic acid. All seawater samples were measured in duplicate.

- Particulate water-extractable organic composition by 1H-NMR spectroscopy in seawater and surface microlayer. All the Quartz-fiber filters collecting Particulate organic carbon (POC) from the seawater (SW) and Surface Micro Layer (SML) were extracted with deionized ultra-pure water (Milli-Q). The extracts were used for water-soluble organic carbon (WSOC) characterization by 1H-NMR spectroscopy as described in Decesari et al. (2020) and here below.

Text S3. SW, SML and Sea-Spray Aerosol (SSA) chemical characterization by 1H-NMR

The POC from the seawater (SW) and Surface Micro Layer (SML) as well as the PM_1 filter samples collected during the bubble-bursting SSA production experiments were extracted in deionized water and analysed by 1H-NMR spectroscopy using the same procedure employed in previous studies (Decesari et al., 2020; Paglione et al., 2024). Briefly, the 1H-NMR spectra were acquired in a 5mm probe using a Varian Unity INOVA 600MHz spectrometer. Sodium 3-trimethylsilyl-(2,2,3,3-d4) propionate (TSP-d4) was used as an internal standard by adding 50 μL of a 0.05% TSP-d4 (by weight) in D2O to the standard in the probe. The extracts were buffered to pH~3 using a deuterated-formate/formic-acid (DCOO- =HCOOH) buffer prior to the analysis in order to avoid the shifting of pH-sensitive signals (Decesari et al., 2024). The 1H-NMR spectroscopy in protic solvents provides speciation of hydrogen atoms bound to carbon atoms. A comprehensive explanation and review of the 1H-NMR applications to environmental samples of atmospheric relevance can be found elsewhere (Decesari et al., 2024; Tagliavini et al., 2024; Paglione et al., 2024). Briefly, on the basis of the range of frequency shifts, the signals in the 1H-NMR spectra can be attributed to specific functional groups containing H–C bounds (Decesari et al., 2000, 2007). The main functional groups identified in this study are the same described in Paglione et al., 2024: unfunctionalized alkyls (H–C, also named “aliphatic chains”); aliphatic protons adjacent to unsaturated/substituted groups (benzyl and acyl groups: H–C–C =) and/or heteroatoms (amines, sulfonates: H–C–X, with X ≠ O), also named “polysubstituted aliphatic chains”; aliphatic hydroxyl/alkoxy groups (H–C–O), also abbreviated later as “Sug–Alc–Eth–Est”; anomeric and vinylic groups (O–CH–O), from sugar/anhydro-sugar derivatives

(glucose, sucrose, levoglucosan, glucuronic acid, etc.); and, finally, aromatic functionalities (Ar–H, also abbreviated later as "Arom"). Organic hydrogen concentrations directly measured by 1H-NMR were converted to organic carbon using stoichiometric ratios specifically assigned to functional groups using the same rationale described in previous works (Decesari et al., 2007; Tagliavini et al., 2006; Paglione et al., 2024).
Moreover, specific organic tracers can be univocally identified in the 1H-NMR spectra on the basis of their characteristic patterns of resonance and chemical shifts. We use for this scope extensive libraries of reference spectra of standard single compounds and/or mixtures from laboratory/chamber experiments and/or from ambient field studies at near-source stations (Paglione et al., 2024). Among all the tracers identified, the most relevant in this work are: lactic acid – Lac; Iso-but: isobutyric acid; Ace: acetic acid; low-molecular-weight alkyl amines (mono- di- and tri-methyl amines, MA, DMA and TMA, respectively); betaine – Bet; choline – Cho; Ac-cho: acetyl-choline; glycerol – Gly; glucose – Gls; sucrose – Suc; and some aminoacids (Tyr, Phe, Glu, Ala, Thr, Leu, Ile, in standard nomenclature). In this study, we also refer to broadly defined chemical classes synonymously with the classes of compounds carrying specific functional groups or combinations of them, like "polyols" (i.e., compounds with NMR bands in the H–C–O region) or "saccharides" (similar to polyols but with the concomitant presence of NMR signals in the anomeric region O–CH–O). Intense NMR bands in the H–C (unfunctionalized alkyls) region with prominent peaks characteristic of aliphatic chains (terminal methyls at 0.9 ppm, methylenic chains at 1.2 ppm, and methines or methylenes in beta position to a C = O group or an oxygen atom at 1.5 ppm) were attributed to compounds from the degradation of lipids (sometimes defined concisely as "lipids") including low-molecular-weight fatty acids (LMW-FA) and mixtures of other alkanoic acids. Our analysis targets the water-soluble fraction of POC while the purely insoluble organic species, such as lipids, are not accounted for. Nevertheless, we employ here the term "lipids" for referring to aliphatic water-soluble compounds exhibiting NMR spectra features characteristic of lipids or compounds of similar chemical structure, e.g. low-molecular weight fatty acids that, in contrast to long chain fatty acids, can be recovered into water solution. Many samples actually showed a pattern of resonances at 1.6, 1.2 (main) and 0.9 ppm of chemical shift which is completely consistent with that of alkanoic acids like Low-Molecular Weight Fatty Acids (LMW-FA). For more details please refer also to Paglione et al. (2024). Therefore, we label such compounds as "LMW-FA" (alkanoic acids) in examining the 1H-NMR spectra and more generally "lipids" when referring to the classes of

compounds they belong, although we could not achieve a molecular-level speciation for them in this study.

Text S4. Factor Analysis of H-NMR POC & SSA Spectra.

During the PI-ICE field study, a number of SW, SML and SSA samples were collected in order to achieve the multiple objectives of the project (Dall´Osto et al., 2022a,b; Zeppenfeld et al., 2020; Brean et al., 2021; Paglione et al., 2024). Here, our main objective is to study the SSA production of different ambient real water collected in situ. Previous experiments reported the study of melting sea ice samples (Dall´Osto et al, 2022a), different leaching materials including guano and kelp forest (Dall´Osto et al., 2022b). Other studies including viral attack and seaweeds leaching experiments are in preparation. During this study, we run 9 experiments with ambient Antarctic water on which the SMPS size distributions was obtained and shown in Figure 1. Given the main objective of this study is to link SSA production, the limiting dataset is the SMPS one, where 9 complete SSA bubble chamber experiment with ambient water were run (Figure 1, Table S1-S3). Nonetheless, for the statistical analysis of the chemical dataset, we used 35 samples for the PCA analysis shown in Figure 2, 46 samples for the factor analysis of the SW-SML samples, and 16 samples for the factor analysis of the SSA samples, as shown in Table S2. The different statistical analyses on the overall PI-ICE datasets (Table S2) allows us to better describe the specific 9 ambient water experiments described in this study (Table S1). In table S3 we summarized the H-NMR identified/measured chemical species/categories and of which factor they are representative.

The original NMR spectra were subjected to several preprocessing steps in order to remove spurious sources of variability prior to the application of factor analysis. A polynomial fit was applied to baselines and subtracted from the spectra. Careful horizontal alignment of the spectra was performed using the Tsp-d4 and buffer singlets as reference positions (at 0.00ppm and 8.45ppm, respectively). The spectral regions containing only noise or sparse signals of solvent/buffer (H< 0.5 ppm; 4.7 < H< 5.2 ppm; and 8.15< H <8.60 ppm) were omitted. Signals associated to blanks (Ar-H at 8.14-8.10, 7.69-7.62, and 7.38-7.36 ppm; vynil-anomeric at 6.43-6.39, 6.20-6.16, and 5.98-5.96ppm; HC-C=O at 2.38-2.36 ppm) were removed because considered not environmentally relevant. Binning over 0.02 ppm of

chemical shift intervals was applied to remove the effects of peak position variability caused by matrix effects. Low-resolution spectra (~400-points) were finally obtained and processed by factor analysis (both PCA and non-negative techniques).

The non-negative factor analysis techniques used in this study include two different algorithms: the "multivariate curve resolution" (MCR), according to the classical alternating least-square approach (Jaumot et al., 2005; Tauler 1995) and the "Positive Matrix Factorization" approach (PMF, Paatero and Tapper, 1994) by applying the Multilinear Engine 2 solver (ME-2, Paatero, 2000) controlled within the Source Finder software (SoFi v4.8, Canonaco et al., 2013; Crippa et al., 2014). Since PMF also requires uncertainties, an uncertainty matrix was derived here from the signal to noise ratios of the NMR spectra (as already described in previous publications): it is calculated as two times the standard deviation of the spectra baseline in a region of the spectra without any signals (i.e., the chemical shift region 6.5-7ppm) (Paglione et al., 2024). Solutions with different number of factors (p= from two up to eight) were explored for both the spectral datasets. The number of factors was chosen for each dataset based on the best separation of interpretable spectral features (compared also with the PCA described in the main text) and of the best agreement between the two algorithms applied with respect to both spectral profiles and contributions.

POC SW-SML dataset.

Regarding the SW-SML dataset, a five-factors (p=5) solution was chosen. The 4-factors solution (p=4) was also considered, but rejected in the end because saccharides were mixed up together with the other constituents such as glycerol, methyl-amines, acrylic acid and mixtures of aliphatic compounds including low-molecular weight fatty acids. Going to 6-factors instead, the solutions start to be less robust producing multiple factors for the same constituents and in disagreement between the two methodologies of factor analysis applied. The interpretation of factor spectral profiles was based on the presence of molecular resonances of tracer compounds, and on the comparison with a library of reference spectra recorded in laboratory or in the field during near-source studies (Facchini et al., 2008; Schmitt-Kopplin et al., 2012; Paglione et al., 2014b; Decesari et al. 2020; Paglione et al., 2024). Fig. S2 and Fig. S3 report profiles and contributions of the H-NMR PMF factors identified by the statistical analyses of the POC and SSA datasets, respectively. In particular, about POC dataset (Fig. S2), the SW-SML Factor 1 is characterized by Glucose

and other sugars resonances and then it is called “Saccharides” factor; SW-SML Factor 2 is mainly characterized by the presence in the spectral profile of common nitrogenated metabolytes such as Betaine, Choline and Acetyl-choline and is then named as “N-osmolytes” factor; SW-SML Factor 3 profile is presenting signals of all the most common aminoacids (such as Alanine, Leucine, Isoleucine, Tyrosine, Threonine, Phenylalanine, etc.) and is therefore named “proteins” factor; SW-SML Factors 4 and 5 exhibit spectral profiles attributable to other mixed constituents like glycerol, methyl-amines, acrylic acid and mixtures of aliphatic compounds including low-molecular weight fatty acids and they are so called “Lipids/ Polyols A” and “Lipids/Polyols B”.

SSA dataset.

Concerning the SSA dataset instead, a two-factors solution was considered already informative enough to describe the variability of the SSA spectra and chosen as the most interpretable solution. The 3- and 4-factors solutions, even if reducing the residuals, generate factors representing single samples instead that common chemical features and for this reason they were considered not useful for the aim of our work, although meaningful for some aspects. About the SSA dataset analysis (Fig. S3), the first factor (SSA Factor 1 – POA lipids-polyols-saccharides) is mainly characterized by aliphatic chains with terminal methyl moieties typical of lipids (bands at 0.9 and 1.3ppm). Together with these aliphatic chains the spectral profile of SSA F1 shows also strong signals in the region of sugars and polyols (e.g., glycerol among others), which are also partially consistent with the spectral features of the SW-SML “Saccharides” factor (SW-SML Factor 1 in Fig. S2). SSA Factor 1 also shows the presence of nitrogen-containing metabolites (betaine) and of low molecular weight amines (dimethylamine, DMA, and traces of monomethylamines and trimethylamines) even if in very minor amounts. It is plausible that betaine, glycerol, and other sugars have a chemical bond to lipids, making glycolipids and phospholipids, which could explain their preferential enrichment during the aerosolization process with respect to other POC constituents like amino acids.

The second Factor (SSA Factor 2 – Lactic Acid) instead, has sparse signals of a different mixture of aliphatic chains and polyols (including again glycerol signals) but is especially characterized by the strong contribution of lactic acid. Compared with the PCA presented in the main text (and in Figure S1), the non-negative factor analysis of the SW-

SML and SSA datasets have strong similarities but is also able to better isolate specific categories and to quantify the components thanks to the non-negativity constrain: SW-SML F2 is overlapping very well the PC1 positive values, while PC2 has specific peaks in common with SW-SML F4 and SSA F2. PC3 and PC4, which are separating the most H-AP and L-AP groups of samples and are so of fundamental importance for the purposes of this work, are instead well splitted by PMF in their original negative part (represented by SW-SML F3 for the proteinaceous fraction and by SW-SML F1 for the saccharides one) and positive part (represented by both SW-SML F4 and especially SW-SML F5 constituted of glycerol, methyl-amines, acrylic acid and mixtures of aliphatic compounds including low-molecular weight fatty acids). Similarly, SSA F1 resemble all the features of the positive part of PC3 and PC4 together.

Text S5 Statistical analysis of the main phytoplankton communities

Moreover, a principal component analysis (Estrada et al., 2016; Legendre and Legendre, 1998) was conducted on the biological dataset to summarize the main trends of the phytoplankton composition in the seawater samples. The analysis was based on the correlation matrix among the log-transformed abundance of 21 taxa (including nanoflagellates from the DAPI counts) that were present in 50% or more of the samples (see supporting information). The expression used for the logarithmic transformation of abundance x was x’ = log(x+c); where c is the smallest abundance recorded in the inverted microscopy (20 cells L-1) or epifluorescence (10000 cells L-1) determinations. The software package used was Systat 13.

Station	Date	Description	Lat. (Deg.)	Lon. (Deg.)	T. (ºC)	Sec.Disk (m)	Sal. (psu)	Sol. Rad (W m-2)	W.S. (m s-1)
**SW3**	26/01/19	Near Avian Island (far from coast)	-67.78	-68.80	1	4	32.4	301	3
**SW4**	26/01/19	Near Avian Island (penguin colony close)	-67.77	-68.87	0.8	5.5	32.4	301	3
**SW6**	28/01/19	Near Bisco Island (penguin colony)	-64.81	-63.77	NA	7	32.0	118	6
**SW7**	29/01/19	Near Primavera Base (marginal ice zone)	-64.15	-60.97	1	8.5	33.0	107	4
**SW8**	29/01/19	Near Primavera Base (marginal ice zone)	-64.15	-60.95	0	7	33.2	107	4
**SW9**	03/02/19	Near Weddell Sea (marginal ice zone)	-63.40	-56.82	NA	NA	33.9	208	9
**SW11**	14/02/19	Near Bahia Sur (Bay)	-62.66	-60.39	NA	NA	NA	58	1
**SW12**	20/02/19	Near Bahia Sur (Bay)	-62.66	-60.39	2.2	3.2	NA	152	1
**SW15**	24/02/19	Near Bahia Sur (Bay)	-62.66	-60.44	2.3	2.5	32.9	112	2

Table S1 Sampling time, location, temperature, secchi disk, salinity of the nine water samples discussed in this paper out the total ones taken during the PI-ICE bubble bursting SSA production experiments (reported on the map in Fig. S1 and Tab. S1). Solar radiation and wind speed data are meteorological data from the sampling day

			available characterization				
			SW	SML	SSA		
Type	Station/sample ID	Decription	NMR-POC	NMR-POC	SMPS-PSD	NMR-SSA	References
Ambient sea-water	SW1	Ambient sea water sample	x	x		x	this work
	SW2	Ambient sea water sample	x	x			this work
	SW3	Ambient H-AP sample	x	x	x	x	this work
	SW4	Ambient L-AP sample	x	x	x	x	this work
	SW5	Ambient sea water sample	x	x			this work
	SW6	Ambient L-AP sample	x	x	x		this work
	SW7	Ambient H-AP sample	x	x	x	x	this work
	SW8	Ambient H-AP sample	x	x	x	x	this work
	SW9	Ambient H-AP sample	x		x	x	this work
	SW11	Ambient L-AP sample			x	x	this work
	SW12	Ambient L-AP sample			x		this work
	SW15	Ambient L-AP sample	x	x	x	x	this work
	SW16	Ambient sea water sample	x				this work
	SW17	Ambient sea water sample	x	x			this work
	SW18	Ambient sea water sample	x				this work
	SW19	Ambient sea water sample	x	x			this work
	SW20	Ambient sea water sample	x	x			this work
	SW21	Ambient sea water sample	x		x		Dall'Osto et al., 2022 b; this work
Perturbed-water Experiments	SW6_AF	Seaweeds leaching	x		x	x	this work; other work on prep.
	ALG1	Seaweeds leaching			x	x	this work; other work on prep.
	ALG2	Seaweeds leaching	x		x	x	this work; other work on prep.
	SW22	Seaweeds leaching	x		x		Dall'Osto et al., 2022b; this work
	SW23	Seaweeds leaching	x		x		Dall'Osto et al., 2022b; this work
	SW24	Seaweeds leaching	x		x		Dall'Osto et al., 2022b; this work
	SW25	Guano leaching	x		x		Dall'Osto et al., 2022b; this work
	SI_1 (t=0)	Melting sea ice sample	x		x	x	This work, Dall'Osto et al., 2022a
	SI_1 (t=24)	Melting sea ice sample	x		x	x	This work, Dall'Osto et al., 2022a
	SI_1E (t=0)	Virus attack experiment	x		x	x	this work; other work on prep.
	SI_1E (t=24)	Virus attack experiment	x		x	x	this work; other work on prep.
	SI_2 (t=0)	Melting sea ice sample	x		x	x	This work, Dall'Osto et al., 2022a
	SI_2 (t=24)	Melting sea ice sample	x		x	x	This work, Dall'Osto et al., 2022a
	SI_2E (t=0)	Virus attack experiment	x		x	x	this work; other work on prep.
	SI_2E (t=24)	Virus attack experiment	x		x	x	this work; other work on prep.
	SI_3 (t=0)	Melting sea ice sample	x		x	x	This work, Dall'Osto et al., 2022a
	SI_3 (t=24)	Melting sea ice sample	x		x	x	this work; other work on prep.
	SI_4 (t=0)	Melting sea ice sample	x		x	x	this work; other work on prep.

Table S2. Short description of all the water samples collected during the PI-ICE and used for bubble bursting SSA production experiments in this work and/or other works (as reported in the last column). For each sample is available the biogeochemical characterization described in Text S2 and summarized in Tab. S3, while here is shown for which samples are available the additional characterization by 1H-NMR and the SSA size distribution characterization by SMPS largely discussed in the present manuscript.

			POC Factor analysis					SSA Factor analysis	
**name of the species/ *category of compounds******	**ID of the species/ functional group**	**chemical shifts used for identification & quantification**	POC F1	POC F2	POC F3	POC F4	POC F5	SSA F1	SSA F2
low-molecular weight amines	LMW amines						X	X	
di-methylamine	DMA	singlet at 2.72 ppm					X		
tri-methylamine	TMA	singlet at 2.89 ppm					X	X	
N-osmolytes		singlets between 3.1 and 3.3		X					
betaine	Bet	singlet at 3.25 ppm		X				X	
choline	Cho	singlet at 3.18 ppm		X					
acetyl choline	Ac-Cho	singlet at 3.20 ppm (not quantified)		X					
aminoacids					X				
tyrosine	Tyr	doublets at 6.9 – 7.2 ppm & specific structures between 3 and 4 ppm (not quantified)			X				
phenylalanine	Phe	specific structures at 7.4 and between 3 - 4 ppm (not quantified)			X				
glutamic acid	Glu	specific multiplets at 3.72-3.74ppm & 2.3-2.4 ppm (not quantified)			X				
alanine	Ala	doublet at 1.48 ppm & quadruplet at 3.73 ppm (not quantified)			X				
threonine	Thr	doublet at 1.32 ppm & specific structures between 3.5 and 4.2 ppm (not quantified)			X				
valine	Val	doublets at 0.9 - 1.1 ppm & specific structures between 2.2 and 3.6 ppm (not quantified)			X				
leucine	Leu	triplets at 0.95 ppm & multiplets at 3.72 and 1.70 ppm (not quantified)			X				
isoleucine	Ile	multiplets at 1.02, 1.25, 1.35, 3.6 ppm (not quantified)			X				
saccharides	Sac	used synonymously for compounds carrying H-C-O groups in unresolved mixtures but when also anomeric protons (O-CH-O) are present	X					X	
glucose	Gls	anomeric doublet at 5.22 ppm & specific structures between 3.5 and 4.2 ppm (not quantified but possibly quantifiable @5.22 ppm)	X					X	
sucrose	Suc	anomeric doublet at 5.40 ppm & specific structures between 3.5 and 4.2 ppm (not quantified but possibly quantifiable @5.40 ppm)	X					X	
polyols		unresolved mixture not quantified (including glycerol and D-threitol)				X	X	X	
glycerol	Gly	specific structures at 3.55, 3.66 & 3.77 ppm (not quantified but possibly quantifiable @ 3.55 ppm)					X	X	X
low-molecular weight fatty acids or "lipids"	LMW-FA	unresolved complex resonances at 0.9, 1.3, and 1.6 ppm in the H-C spectral region				X	X	X	
acrylic acid	Acr	multiplets between 5.8 and 6.4ppm					X	X	
acetic acid	Ace	singlet at 2.06 ppm (not quantified)				X			
isobutyric acid	Iso-but	doublet at 1.12-1.13 ppm & specific structures between 2.4 ppm (not quantified)			X	X			
lactic acid	Lac	doublet 1.37-1.36 ppm & quadruplet at 4.23 ppm (not quantified but possibly quantifiable @1.37-1.36 ppm)				X			X

Table S3. H-NMR identified/measured chemical species/categories. *Categories including some of the other species specifically identified are in *underlined italic*

Units	Group 1 (far from land, blue points, N=4) Mean ± St dev	Group 2 (close to land, orange points, N=5) Mean ± St dev
**Biogeochemical components**		
POC (µM)	18.7 ± 12.0	32.1 ± 12.5
DOC (µM)	55.1 ± 5.0	61.8 ± 15.6
PN (µM)	3.2 ± 2.4	4.7 ± 1.9
TN (µM)	27.6 ± 8.0	26.2 ± 2.4
C/N (µM/µM)	6.9 ± 2.6	6.8 ± 0.7
TEP (XG eq. µg L-1)	***28.7 ± 8.9***	***103.9 ± 63.2***
CSP (BSA eq. µg L-1)	***39.2 ± 32.0***	***89.6 ± 44.3***
DMSP (nM)	67.2 ± 38.5	153.4 ± 89.3
**Nutrients**		
Nitrate (µM)	22.8 ± 5.5	22.8 ± 2.5
Ammonia (µM)	0.5 ± 0.7	0.8 ± 0.7
Silicate (µM)	69.7 ± 10.8	59.6 ± 16.3
Phosphate (µM)	1.6 ± 0.5	1.5 ± 0.3
**Microbiota**		
Virus, V1 (virus/mL)	6.74E+05 ± 8.15E+04	1.29E+06 ± 2.66E+05
Virus, V2 (virus/mL)	8.07E+05 ± 1.80E+05	1.32E+06 ± 5.31E+05
Virus, V3 (virus/mL)	4.07E+04 ± 4.45E+03	6.19E+04 ± 3.38E+04
Virus, V4 (virus/mL)	1.46E+05 ± 3.86E+04	4.77E+05 ± 3.97E+05
Virus Total (virus/mL)	1.67E+06 ± 2.35E+05	3.15E+06 ± 7.11E+05
Virus Total biomass (µg C/L)	0.3 ± 0.0	0.6 ± 0.1
Total Prokaryote (cell/mL)	3.63E+05 ± 2.50E+05	5.51E+05 ± 2.93E+05
HDNA Prokaryote (cell/mL)	2.58E+05 ± 2.21E+05	3.55E+05 ± 2.37E+05
LDNA Prokaryote (cell/mL)	8.92E+04 ± 4.66E+04	1.67E+05 ± 7.26E+04
Total biomass Prokaryote (µg C/L)	6.5 ± 4.5	9.9 ± 5.3
HDNA biomass Prokaryote (µg C/L)	5.0 ± 4.3	7.0 ± 4.6
LDNA biomass Prokaryote (µg C/L)	1.4 ± 0.7	2.6 ± 1.1
Chl-a (µg/L)	1.4 ± 2.4	4.1 ± 4.5
HNF abundance (cell/mL)	1219 ± 784.2	1153 ± 643.1
HNF biomass (µg C/L)	20 ± 14.1	19.8 ± 10.4
HNF ≤2µm (cell/mL)	139 ± 71.8	234.4 ± 227.9
HNF 2-5µm (cell/mL)	608 ± 468.5	508.1 ± 251.8
HNF 5-10µm (cell/mL)	420 ± 325.0	353.6 ± 223.8
HNF 10-20µm (cell/mL)	50 ± 66.4	56.9 ± 47.2
PNF abundance (cell/mL)	1960 ± 1573.0	6715.0 ± 8469.1
PNF biomass (µg C/L)	66 ± 73.3	38.7 ± 32.7
PNF ≤2µm (cell/mL)	250 ± 188.8	290.5 ± 236.7
PNF 2-5µm (cell/mL)	1262 ± 1133.3	6054.8 ± 8151.1
PNF 5-10µm (cell/mL)	120 ± 96.4	286.9 ± 332.0
PNF 10-20µm (cell/mL)	333 ± 395.7	82.8 ± 52.8

Table S4. Marine biogeochemical variables collected and the two groups of samples generating H-AP and L-AP concentrations. Only TEP and CSP (in bold) shows statistically different averages at two sigma.

r (Pearson)					
NMR-Factors	F1	F2	F3	F4	F5
Chem. Groups	Saccharides	N-osmolytes	Proteins	lipids/polyols A	lipids/polyols B
Virus	0.19	0.25	0.23	-0.12	-0.09
Bacteria	0.11	0.13	0.50	-0.15	-0.11
POC	0.90	0.96	0.76	-0.17	-0.19
DOC	0.81	0.88	0.67	-0.21	-0.10
TEP	0.68	0.71	0.73	-0.18	-0.23
CSP	0.93	1.00	0.75	-0.17	-0.17
FDOM Biol. index	0.18	0.17	0.18	0.21	-0.20
FDOM Peak B	0.83	0.86	0.84	-0.24	-0.14
FDOM Peak T	0.80	0.87	0.70	-0.22	-0.15
FDOM Peak A	0.72	0.77	0.63	-0.23	-0.10
FDOM Peak M	0.66	0.69	0.60	-0.22	-0.06
FDOM Peak C	0.59	0.59	0.55	-0.22	0.01
FDOM Fluor. Index	0.17	0.14	0.12	0.10	0.01
FDOM Humific. Index	-0.29	-0.33	-0.35	0.21	0.05
FDOM A254	0.04	0.05	0.01	-0.18	0.10
Fucose	0.11	0.45	-0.10	0.06	0.04
Galactosamine	0.47	0.54	0.20	0.02	-0.01
Rhamnose	0.14	-0.16	-0.24	0.10	-0.12
Arabinose	0.04	0.04	-0.30	0.13	0.00
Glucosamine	0.31	0.61	0.12	0.05	0.03
Galactose	0.67	0.27	0.21	-0.02	-0.10
Glucose	0.73	-0.20	0.17	0.03	-0.24
Xylose	0.48	0.32	0.03	0.08	-0.02
Mannose	0.06	0.14	-0.07	0.03	-0.02
Muramic acid	0.94	-0.13	0.32	-0.02	-0.19
Galacturonic acid	0.30	0.17	0.09	0.11	-0.13
Glucuronic acid	0.88	0.00	0.20	0.16	-0.19
DCCHO total	0.74	-0.27	0.06	0.19	-0.23
PCCHO total	0.70	-0.07	0.15	0.04	-0.20

Table S5. Correlation (r, Pearson coefficients) between categories of compounds identified by H-NMR PMF analysis and the chemical/biological composition of the sea-water/sea-ice samples. Single carbohydrates are reported as sum of DCCHO and PCCHO (combined, see supplementary text). The H-NMR factors are named upon the main chemical constituents identified in their spectral profile. Factors F4 and F5 exhibit spectral profiles attributable to other constituents like glycerol, methyl-amines, acrylic acid and mixtures of aliphatic compounds including low-molecular weight fatty acids (see Supplementary material, Figure S1). Colors represents the statistical significance (white is no significance, and dark red is significance at 95% two sigma, all r above 0.55 are statistically significant at two sigma).

Code	Name
Gyrod. spp. H	*Gyrodinium* spp. heterotrophic
Dinofl. L (Un.)	Unidentified dinoflagellates (large)
Dinofl. S (un.)	Unidentified dinoflagellates (< 20 µm)
Amp. sp. 1	*Amphora* sp. 1
Corethron pen.	*Corethron pennatum*
Frag. cyl.	*Fragilariopsis cylindrus*
Frag. spp.	*Fragilariopsis* spp.
Licmoph. spp.	*Licmophora spp.*
Pse.nitz. spp.	*Pseudo-nitzschia* spp.
Thal. S	*Thalassiosira* spp., small (<20 µm Ø)
Thal. M	*Thalassiosira* spp., medium-sized (20-50 µm Ø)
Thal. L	*Thalassiosira* spp., large (>50µm Ø)
Pen sp. 1	Pennate sp. 1
Pen. Diat. S (Un.)	Unidentified pennate diatoms (<20 µm Ø)
Pen. Diat. L (Un.)	Unidentified pennate diatoms, large.
Ciliates (<30 µm)	Ciliates (<30 µm)
HNF Total	Heterotrophic nanoflagellates (HNF)
PNF-Cryp.	Cryptophytes
PNF-Phae.	*Phaeocystis* sp.
PNF 2-5 µm	Unid. phototrophic nanoflagellates, small (PNF 2-5 µm)
PNF 5-20 µm	Unid. phototrophic nanoflagellates, large (PNF 5-20 µm)

Table S6. Coding for the 21 selected phytoplankton variables

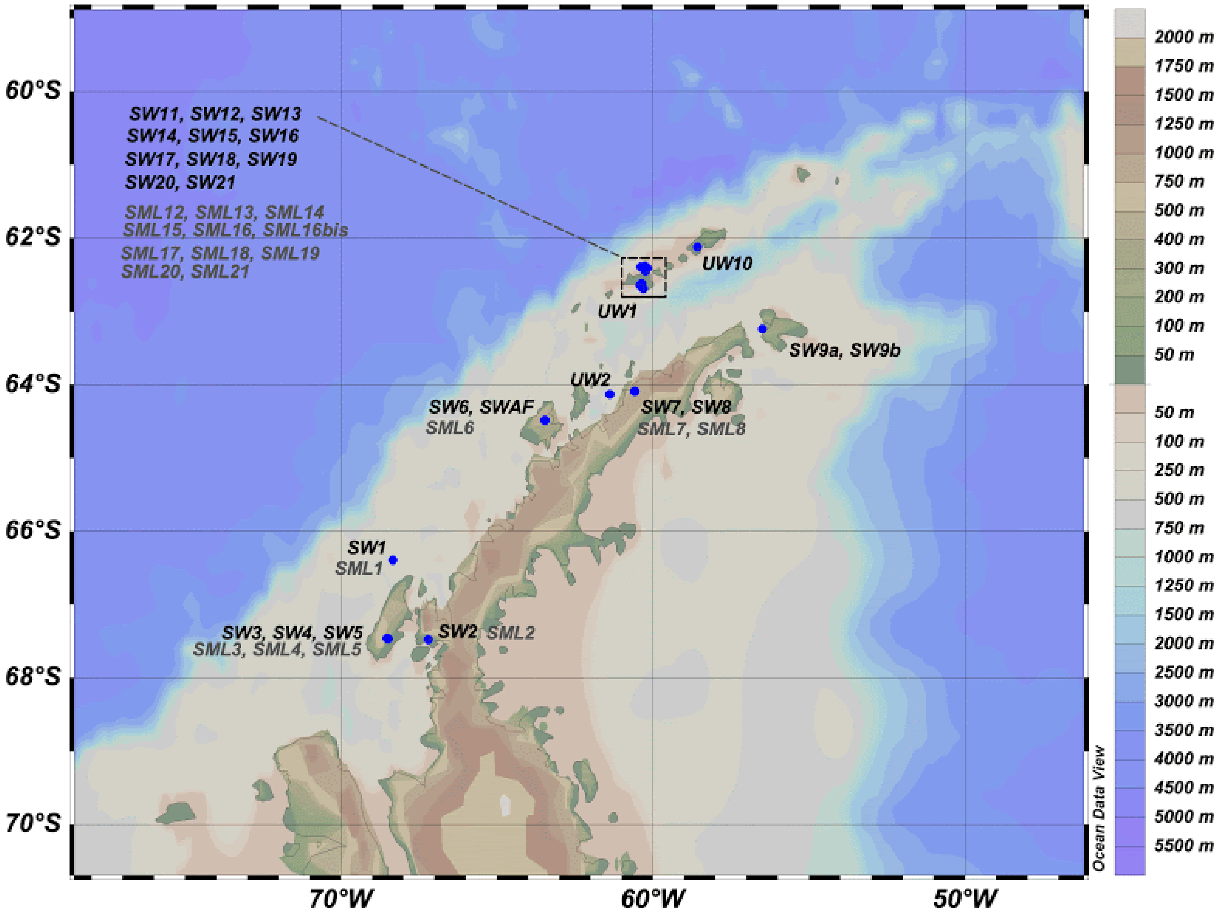

Figure S1. Map of the study Area with SW and SML sampling locations. UW (Under Way) are waters collected from the continuous system of the BIO Hesperides at 7m depth. Here, we only consider SW collected on the surface of the ocean (nine samples described in Table S1).

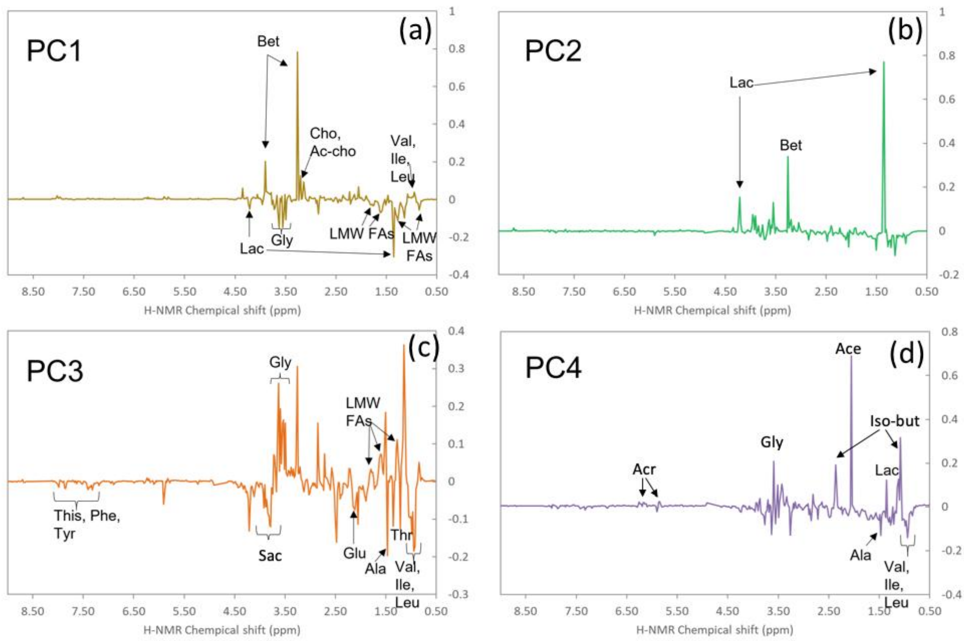

Figure S2. PCA loadings of the PCA H-NMR analysis for PC1 (S2a, top left), PC2 (S2b, top right), PC3 (S2c, bottom left) and PC4 (S2d, bottom right) . H-NMR peaks of aminoacids (Tyr, Phe, Glu, Ala, Thr, Leu, Ile in standard nomenclature) or of other individual compounds (Bet: betaine; Acr: acrylic acid; Lac: lactic acid; Cho: choline; Ac-cho: acetyl-choline; Gly: glycerol; Iso-but: isobutyric acid; Ace: acetic acid) are specified in the loading profiles, along with the band of unresolved mixtures: LMW-FAs (low-molecular weight fatty acids) and Sac (saccharides).

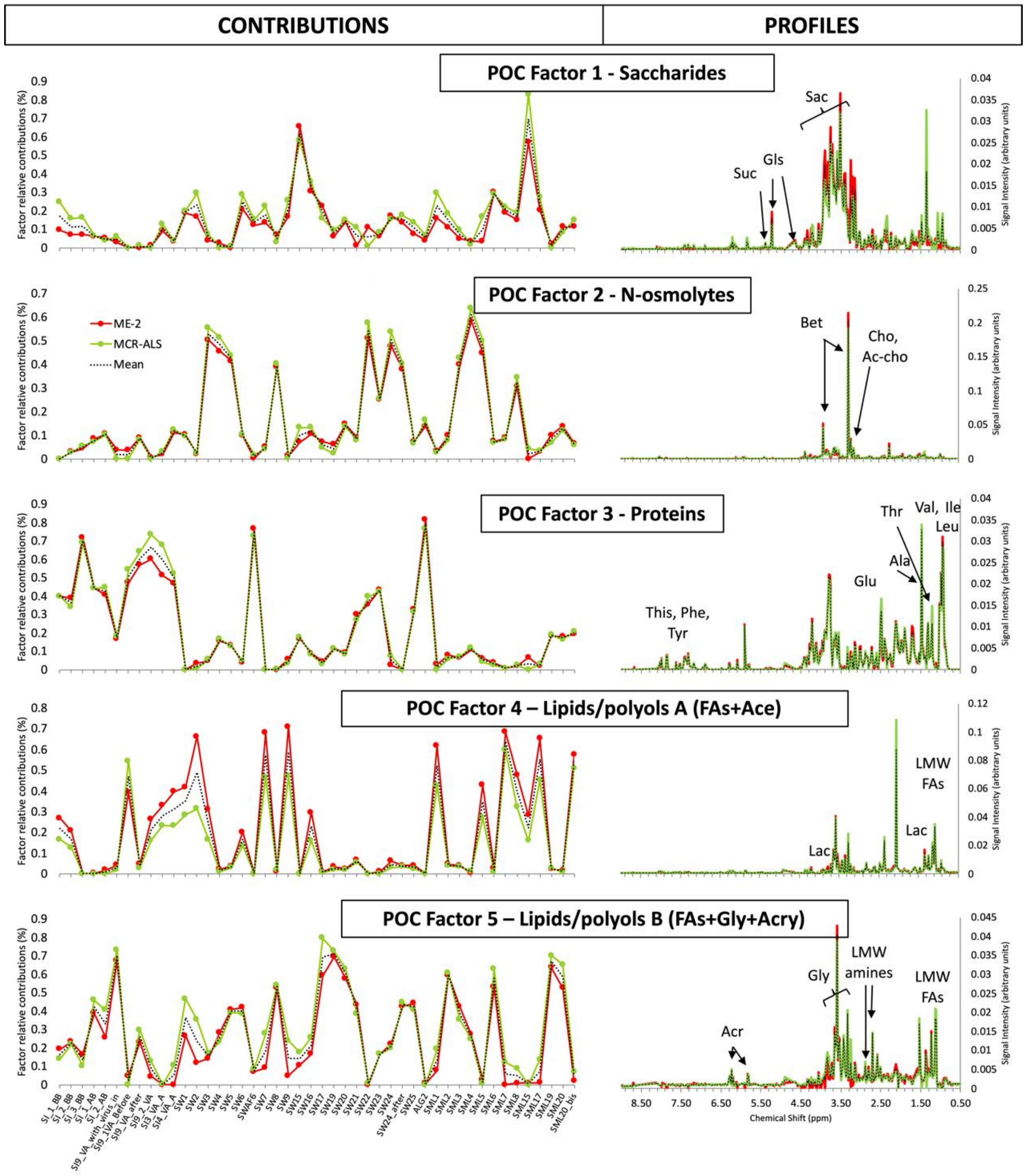

Figure S3. PMF ME-2 and MCR-ALS profiles and contributions of the 5-factors solution from POC dataset of NMR spectra factor analysis. Results from the two different algorithms and the average between them are reported: PMF ME-2 (red line), MCR-ALS (green line), and average value (black line) in each graph. H-NMR peaks of aminoacids (Tyr, Phe, Glu, Ala, Thr, Leu, Ile in standard nomenclature) or of other individual compounds (Bet: betaine; Acr: acrylic acid; Lac: lactic acid; Cho: choline; Ac-cho: acetyl-choline; Gly: glycerol; Gls: glucose; Suc: Sucrose) are specified in the profiles, along with the band of unresolved mixtures: LMW-FAs (low-molecular weight fatty acids), LMW amines (low molecular-weght alkyl amines, such as DMA and TMA) and Sac (saccharides).

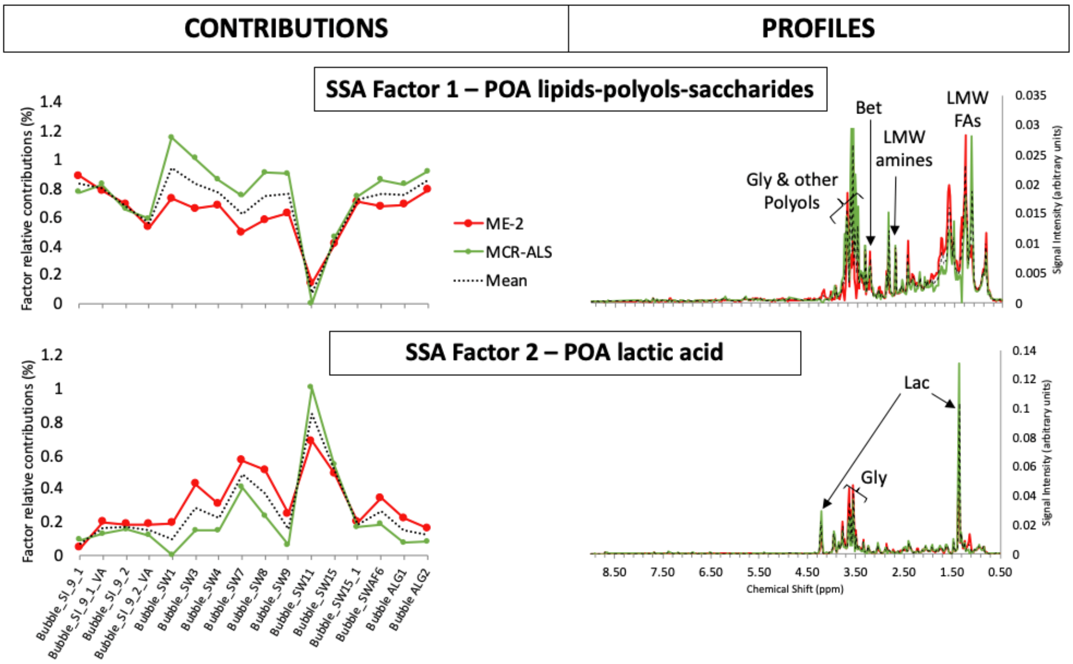

Figure S4. PMF ME-2 and MCR-ALS profiles and contributions of the 2-factors solution from SSA dataset of NMR spectra factor analysis. Results from the two different algorithms and the average between them are reported: PMF ME-2 (red line), MCR-ALS (green line), and average value (black line) in each graph. H-NMR peaks of individual compounds (Bet: betaine; Acr: acrylic acid; Lac: lactic acid; Gly: glycerol) are specified in the profiles, along with the band of unresolved mixtures: LMW-FAs (low-molecular weight fatty acids), LMW amines (low molecular-weght alkyl amines, such as DMA) and polyols.

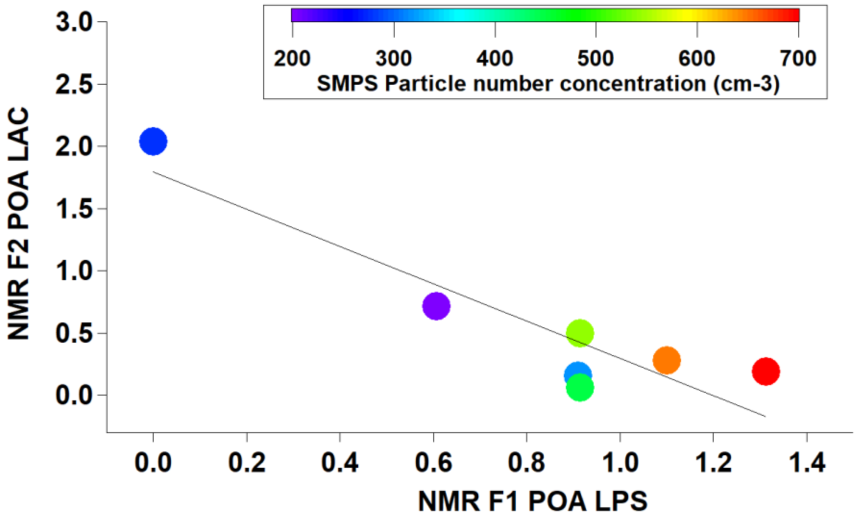

Figure S5 PMF results of the NMR PMF F1 (POA Lipid-polyols-saccharides) and NMR PMF F2 (POA Lactic Acid) for the chemically characterized PM_1 samples, along with the particle number concentrations of each experiments (collected by means of SMPS, 15-500 nm, concentrations in cm^{-3}).

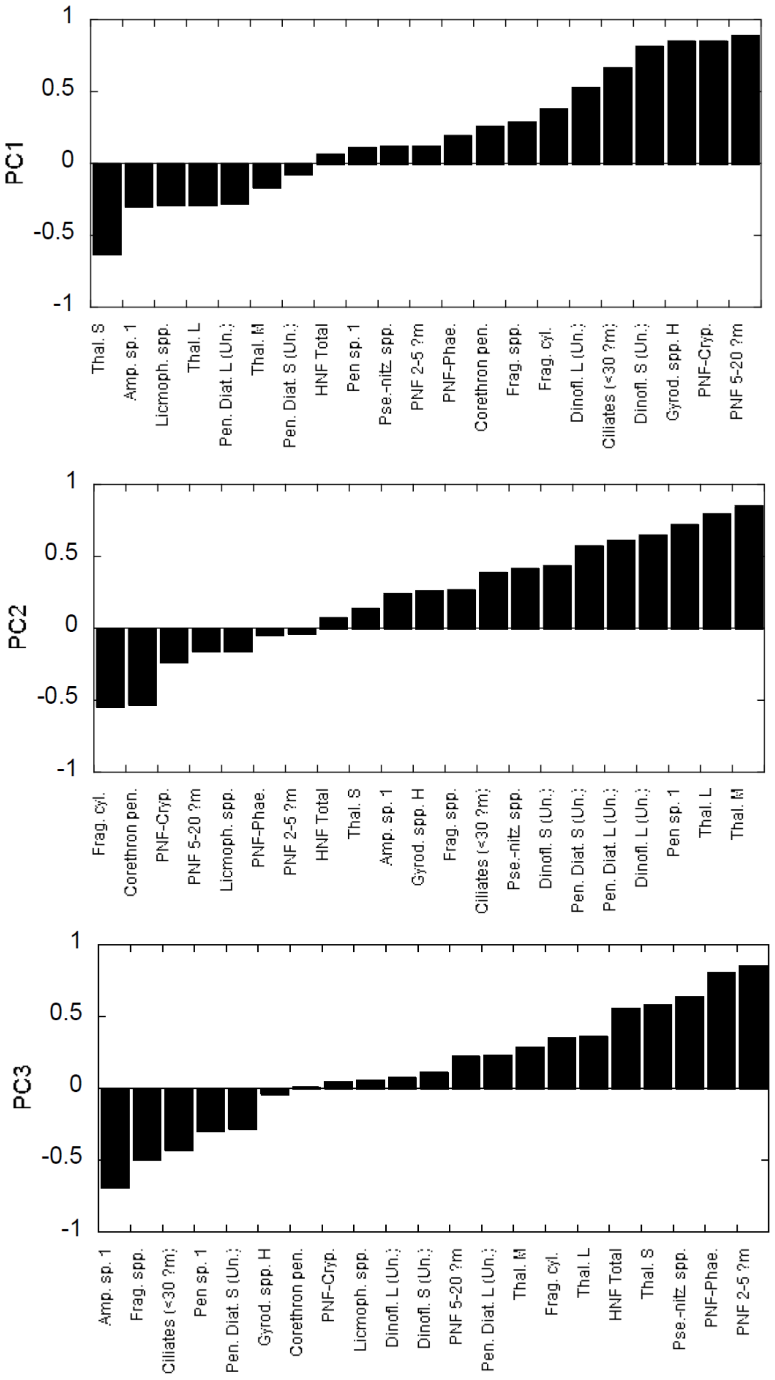

Figure S6. PCA results. Loadings of PC1, PC2 and PC3 on the 21 selected phytoplankton variables, sorted in ascending order (codes are shown in Table S5).

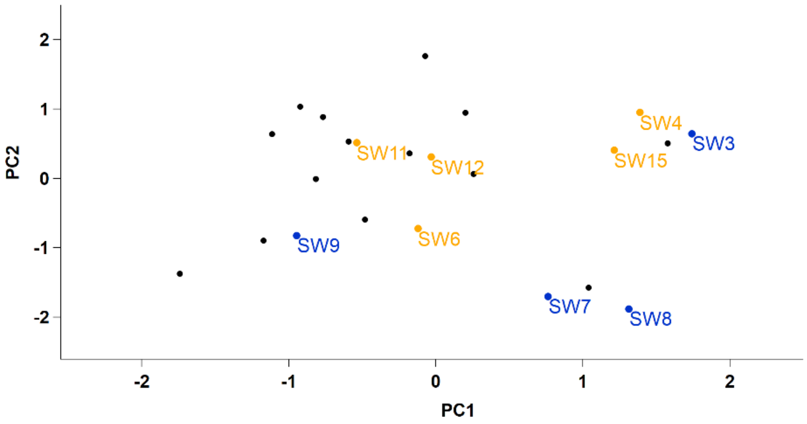

(a)

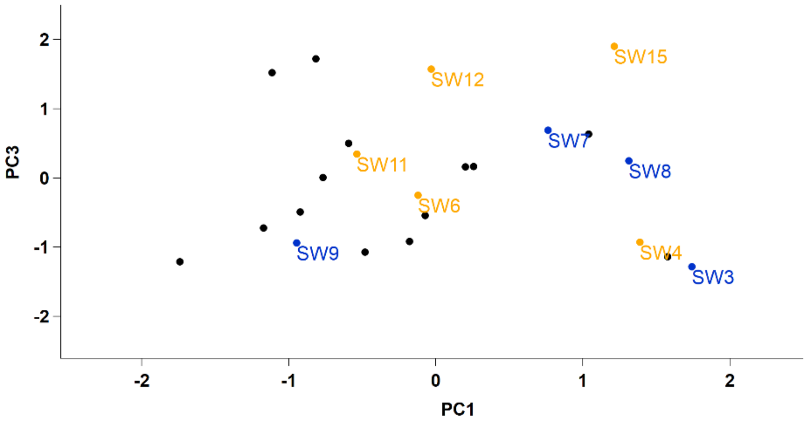

(b)

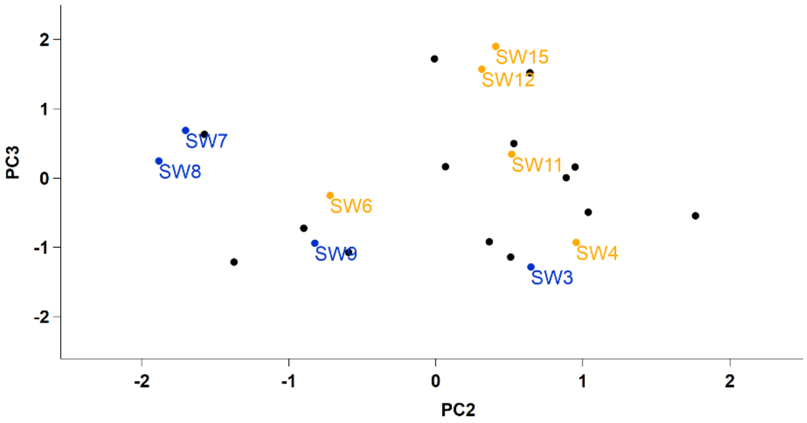

(c)

Figure S7 PCA plots of PC1 vs PC2 (a), PC1 vs PC3 (b) and PC2 vs PC3 (c) for the 21 phytoplankton variables selected.

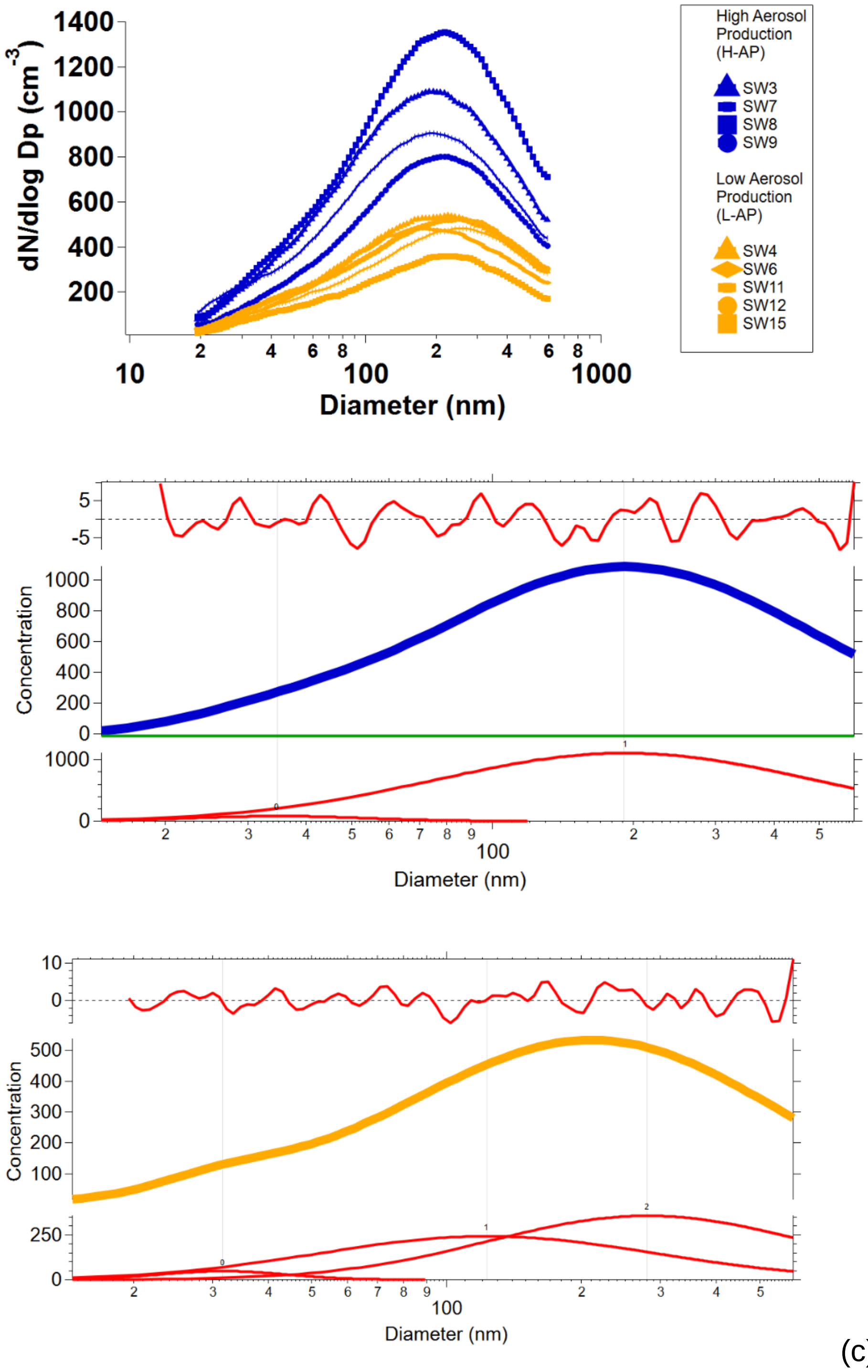

(a)

(b)

(c)

Figure S8 Figure S8a shows the same PNSD presented in Figure 1a, but not in Y log mode. Peak fitting of the two PSD groups seen in Figure 1 (average aerosol size resolved particle number concentrations for bubble bursting SSA production experiments as L-AP and H-AP - Low and High SSA production, as in S8b and S8c). Figures S8b and S8c are described as : peak fitting results (bottom chart), original data (middle chart) and error in the fitting (top chart).

SUPPORTING INFORMATION REFERENCES

Boras, J.A., Sala, M.M., Arrieta, J.M., Sa, E.L., Felipe, J., Agustí, S., Duarte, C.M., Vaqué, D., 2010. Effect of ice melting on bacterial carbon fluxes channelled by viruses and protists in the Arctic Ocean. Pol Biol 33, 1695–1707.

Canonaco, F., Crippa, M., Slowik, J. G., Baltensperger, U., and Prévôt, A. S. H.: SoFi, an IGOR-based interface for the efficient use of the generalized multilinear engine (ME-2) for the source apportionment: ME-2 application to aerosol mass spectrometer data, Atmos. Meas. Tech., 6, 3649–3661, https://doi.org/10.5194/amt-6-3649-2013, 2013.

Cisternas-Novoa, C., Lee, C., Engel, A., 2014. A semi-quantitative spectrophotometric, dyebinding assay for determination of coomassie blue stainable particles. Limnol. Oceanogr.
Methods 12 (AUG), 604–616. https://doi.org/10.4319/lom.2014.12.604.

Crippa, M., Canonaco, F., Lanz, V. A., Äijälä, M., Allan, J. D., Carbone, S., Capes, G., Ceburnis, D., Dall'Osto, M., Day, D. A., De-Carlo, P. F., Ehn, M., Eriksson, A., Freney, E., Hildebrandt Ruiz, L., Hillamo, R., Jimenez, J. L., Junninen, H., Kiendler-Scharr, A., Kortelainen, A.-M., Kulmala, M., Laaksonen, A., Mensah, A. A., Mohr, C., Nemitz, E., O'Dowd, C., Ovadnevaite, J., Pandis, S. N., Petäjä, T., Poulain, L., Saarikoski, S., Sellegri, K., Swietlicki, E., Tiitta, P., Worsnop, D. R., Baltensperger, U., and Prévôt, A. S. H.: Organic aerosol components derived from 25 AMS data sets across Europe using a consistent ME-2 based source apportionment approach, Atmos. Chem. Phys., 14, 6159–6176, https://doi.org/10.5194/acp-14-6159-2014, 2014

Dall'Osto M, Vaque´ D,Sotomayor-Garcia A,Cabrera-Brufau M, Estrada M, Buchaca T, Soler M, Nunes S,Zeppenfeld S, van Pinxteren M,Herrmann H, Wex H, Rinaldi M,Paglione M, Beddows DCS,Harrison RM and Berdalet E (2022)Sea Ice Microbiota in the Antarctic Peninsula Modulates Cloud-RelevantSea Spray Aerosol Production. Front. Mar. Sci. 9:827061.doi: 10.3389/fmars.2022.827061 2022a

Dall'Osto, Manuel, Ana Sotomayor-Garcia, Miguel Cabrera-Brufau, Elisa Berdalet, Dolors Vaqué, Sebastian Zeppenfeld, Manuela van Pinxteren, Hartmut Herrmann, Heike Wex, Matteo Rinaldi, Marco Paglione, David Beddows, Roy Harrison, Conxita Avila, Rafael P. Martin-Martin, Jiyeon Park, Andrés Barbosa, Leaching material from Antarctic seaweeds and penguin guano affects cloud-relevant aerosol production, Science of The Total Environment, Volume 831, 2022, 154772, ISSN 0048-9697, https://doi.org/10.1016/j.scitotenv.2022.154772. 2022b

Decesari, S., Finessi, E., Rinaldi, M., Paglione, M., Fuzzi, S., Stephanou, E. G., Tziaras, T., Spyros, A., Ceburnis, D., O'Dowd, C., Dall'Osto, M., Harrison, R. M., Allan, J., Coe, H., Facchini, M. C.: Primary and secondary marine organic aerosols over the North Atlantic Ocean during the MAP experiment, J. Geophys. Res., 116, D22210, doi:10.1029/2011JD016204, 2011.

Decesari, S., Paglione, M., Rinaldi, M., Dall'Osto, M., Simó, R., Zanca, N., Volpi, F., Facchini, M. C., Hoffmann, T., Götz, S., Kampf, C. J., O'Dowd, C., Ceburnis, D., Ovadnevaite, J., and Tagliavini, E.: Shipborne measurements of Antarctic submicron organic aerosols: an NMR perspective linking multiple sources and bioregions, Atmos. Chem. Phys., 2020, 20, 4193–4207, https://doi.org/10.5194/acp-20-4193-2020

Facchini, M. C., Rinaldi, M., Decesari, S., Carbone, C., Finessi, E.,Mircea, M., Fuzzi, S., Ceburnis, D., Flanagan, R., Nilsson, E. D.,de Leeuw, G., Martino, M., Woeltjen, J., and O'Dowd, C. D.: Primary submicron marine aerosol dominated by insoluble organic colloids and aggregates, Geophys. Res. Lett., 35, L17814, https://doi.org/10.1029/2008GL034210, 2008.

Gasol, J.M., Del Giorgio, P.A., 2000. Using flow cytometry for counting natural planktonic bacteria and understanding the structure of planktonic bacterial communities. Sci. Mar. 64 (2), 197–224.

Holm-Hansen, O., Lorenzen, C.J., Holmes, R.W., Strickland, J.D.H., 1965. Fluorometric determination of chlorophyll. ICES J. Mar. Sci. 30 (1), 3–15. https://doi.org/10.1093/icesjms/30.1.3.

Jaumot, J., Gargallo, R. de Juan, A., and Romà Tauler, R.: A graphical user-friendly interface for mcr-als: a new tool for multivariate curve resolution in matlab. Chemometrics and Intelligent Laboratory Systems, 76(1), 101–110, https://doi.org/10.1016/j.chemolab.2004.12.007, 2005

Li, G., A. Welti, A. Rocchi, G. P. Fogwill, M. Dall'Osto and Z. A. Kanji, Terrestrial and Marine Sources of Ice Nucleating Particles in the Eurasian ArcticFaraday Discuss., 2024, DOI: 10.1039/D4FD00160E.

Paatero, P. and Tapper, U.: Positive matrix factorization: A non-negative factor model with optimal utilization of error estimates of data values, Environmetrics, 5, 111–126, doi:10.1002/env.3170050203, 1994.

Paatero, P.: User's guide for the multilinear engine program "ME2" for fitting multilinear and quasimultilinear models, University of Helsinki, Finland, 2000.

Finessi, E., Decesari, S., Paglione, M., Giulianelli, L., Carbone, C.,Gilardoni, S., Fuzzi, S., Saarikoski, S., Raatikainen, T., Hillamo,R., Allan, J., Mentel, Th. F., Tiitta, P., Laaksonen, A., Petäjä,T., Kulmala, M., Worsnop, D. R., and Facchini, M. C.: Determinationof the biogenic secondary organic aersol fraction in the boreal forest by NMR spectroscopy, Atmos. Chem. Phys., 12, 941–959, doi:10.5194/acp-12-941-2012, 2012.

Paglione, M., Kiendler-Scharr, A., Mensah, A. A., Finessi, E., Giulianelli, L., Sandrini, S., Facchini, M. C., Fuzzi, S., Schlag, P., Piazzalunga, A., Tagliavini, E., Henzing, J. S., and Decesari, S.: Identification of humic-like substances (HULIS) in oxygenated organic aerosols using NMR and AMS factor analyses and liquid chromatographic techniques, Atmos. Chem. Phys., 14, 25–45, https://doi.org/10.5194/acp-14-25-2014, 2014a.

Paglione, M., Saarikoski, S., Carbone, S., Hillamo, R., Facchini, M. C., Finessi, E., Giulianelli, L., Carbone, C., Fuzzi, S., Moretti, F., Tagliavini, E., Swietlicki, E., Eriksson Stenström, K., Prévôt, A. S. H., Massoli, P., Canaragatna, M., Worsnop, D., and Decesari, S.: Primary and secondary biomass burning aerosols determined by proton nuclear magnetic resonance (1H-NMR) spectroscopy during the 2008 EUCAARI campaign in the Po Valley (Italy), Atmos. Chem. Phys., 14, 5089–5110, https://doi.org/10.5194/acp-14-5089-2014, 2014b.

Passow, U., Alldredge, A.L., 1995. A dye-binding assay for the spectrophotometric measurement of transparent exopolymer particles (TEP). Limnol. Oceanogr. 40, 1326–1335. https://doi.org/10.4319/lo.1995.40.7.1326.

Rocchi, A., von Jackowski, A., Welti, A., Li, G., Kanji, Z. A., Povazhnyy, V., Engel, A., Schmale, J., Nenes, A., Berdalet, E., Simó, R., and Dall'Osto, M.: Glucose Enhances Salinity-Driven Sea Spray Aerosol Production in Eastern Arctic Waters, Environ. Sci. Technol., 58, 8748–8759, https://doi.org/10.1021/acs.est.4c02826, 2024

Sieracki, M.E., Johnson, P.W., Sieburth, J.M., 1985. Detection, enumeration, and sizing of planktonic bacteria by image-analyzed epifluorescence microscopy. Appl. Environ. Microbiol. 49 (4), 799–810

Tagliavini E, Decesari S, Paglione M, Mazzanti A, NMR spectroscopic applications to atmospheric organic aerosol analysis – Part 2: A review of existing methodologies and perspectives, TrAC Trends in Analytical Chemistry Volume 172, March 2024, 117595

Tauler R.: Multivariate Curve Resolution applied to second order data, Chem. Int. Laborat. Syst., 30, 133–146, 1995

Zeppenfeld, S., van Pinxteren, M., Engel, A., Herrmann, H., 2020. A Protocol for Quantifying Mono- and Polysaccharides in Seawater and Related Saline Matrices by Electro-dialysis (ED) – Combined With HPAEC-PAD. 16, pp. 817–830. https://doi.org/10.5194/os-16-817-2020.

Zeppenfeld, S., van Pinxteren, M., van Pinxteren, D., Wex, H., Berdalet, E., Vaqué, D., Dall'Osto, M., Herrmann, H., 2021. Aerosol marine primary carbohydrates and atmospheric transformation in the Western Antarctic Peninsula. ACS Earth Space Chem. 5, 1032–1047. https://doi.org/10.1021/acsearthspacechem.0c00351.
